# Supplementary material for: A machine learning approach to evaluate the state of hypertension care coverage: From 2016 STEPs survey in Iran
Source: PLoS One. 2022 Sep 21;17(9):e0273560. doi: 10.1371/journal.pone.0273560 (PMC9491523; doi:10.1371/journal.pone.0273560)

A machine learning approach to evaluate the state of hypertension care coverage: From 2016 STEPs survey in Iran

Supplementary material

Table of contents

Supplemental Methods ... 3

Hyperparameter tuning ... 5

Supplemental Figures ... 13

Supplemental figure S1 – Interactions between age, sex, and area of residence for screening ... 13

Supplemental figure S2 – Interactions between age, sex, and area of residence for diagnosis ... 14

Supplemental figure S3 – Interactions between age, sex, and area of residence for treatment ... 15

Supplemental figure S4 – Interactions between age, sex, and area of residence for control ... 16

Supplemental figure S5 – Interactions between aera of residence, education, and wealth index for screening ... 17

Supplemental figure S6 – Interactions between aera of residence, education, and wealth index for diagnosis ... 18

Supplemental figure S7 – Interactions between aera of residence, education, and wealth index for treatment ... 19

Supplemental figure S8 – Interactions between aera of residence, education, and wealth index for control ... 20

Supplemental figure S9 – Interactions between age, sex, education, and wealth index for screening ... 21

Supplemental figure S10 – Interactions between age, sex, education, and wealth index for diagnosis ... 22

Supplemental figure S11 – Interactions between age, sex, education, and wealth index for treatment ... 23

Supplemental figure S12 – Interactions between age, sex, education, and wealth index for control ... 24

Supplemental figure S13 – Interactions between age, sex, marital status, and wealth index for screening ... 25

Supplemental figure S14 – Interactions between age, sex, marital status, and wealth index for diagnosis ... 26

Supplemental figure S15 – Interactions between age, sex, marital status, and wealth index for treatment ... 27

Supplemental figure S16 – Interactions between age, sex, marital status, and wealth index for control ... 28

Supplemental figure S17 – Interactions between age, sex, area of residence, and marital status for screening ... 29

Supplemental figure S18 – Interactions between age, sex, area of residence, and marital status for diagnosis ... 30

Supplemental figure S19 – Interactions between age, sex, area of residence, and marital status for treatment ... 31

Supplemental figure S20 – Interactions between age, sex, area of residence, and marital status for control 32
Supplemental figure S21 – Interactions between age, sex, body mass index, and diabetes mellitus for screening .. 33
Supplemental figure S22 – Interactions between age, sex, body mass index, and diabetes mellitus for diagnosis .. 34
Supplemental figure S23 – Interactions between age, sex, body mass index, and diabetes mellitus for treatment .. 35
Supplemental figure S24 – Interactions between age, sex, body mass index, and diabetes mellitus for control 36
Supplemental figure S25 – Interactions between age, sex, body mass index, and smoking for screening 37
Supplemental figure S26 – Interactions between age, sex, body mass index, and smoking for diagnosis 38
Supplemental figure S27 – Interactions between age, sex, body mass index, and smoking for treatment 39
Supplemental figure S28 – Interactions between age, sex, body mass index, and smoking for control 40

Supplemental Methods

Definition of the care cascade:

- Definition of the population with HTN
  - Systolic blood pressure ≥ 140 mmHg OR diastolic blood pressure ≥ 90 mmHg
    - Three measurements of blood pressure were taken according to the STEPs protocol. If all three were available in the dataset, the first one was discarded and the average of the latter two was calculated. If two measurements were available, the first one was discarded and the second one was used.
  - Use of anti-hypertensive medications
    - Positive response to either of the questions below:
      - "Are you currently taking medications to lower your blood pressure?"
      - "Has a physician or other health care provider ever prescribed medication for you to reduce your blood pressure?"
  - Previous diagnosis of hypertension
    - Positive response to the question below:
      - "Has a physician or other health care provider ever told you that you have hypertension (high blood pressure)?"
- Step 1, screening
  - Positive response to the question below:
    - "Has a physician or other health care provider ever measured your blood pressure?"
- Step 2, diagnosis
  - Positive response to the question below:
    - "Has a physician or other health care provider ever told you that you have hypertension (high blood pressure)?"

- Step 3, treatment
    - Positive response to either of the questions below:
        - “Are you currently taking medications to lower your blood pressure?”
        - “Has a physician or other health care provider ever prescribed medication for you to reduce your blood pressure?”
- Step 4, control
    - Systolic blood pressure < 130 mmHg AND diastolic blood pressure < 80 mmHg/

It should be noted that reaching each step was conditional on having reached the previous steps.

Hyperparameter tuning

Screening step

mtry	min.node.size	sample.fraction	Accuracy	Kappa
6	5	0.75	0.7950759251	0.5762933287
6	10	0.75	0.7946336429	0.5744337276
6	5	1	0.7940439334	0.5747336377
10	5	0.75	0.7937490786	0.5731967749
6	10	1	0.7937490786	0.5730106824
6	1	0.5	0.793159369	0.5708917726
6	1	0.75	0.793159369	0.5715656764
6	5	0.5	0.7916850951	0.5686248855
6	5	0.25	0.7915376677	0.5682254406
10	5	1	0.7913902403	0.5687665985
6	1	1	0.7910953855	0.5678937051
10	1	0.25	0.7908005307	0.5659223004
10	5	0.5	0.7903582486	0.5658583391
10	1	0.5	0.7902108212	0.5664040395
6	10	0.5	0.7888839746	0.5622325094
10	1	0.75	0.7884416925	0.5632038962
10	10	0.5	0.7875571281	0.5597695631
10	5	0.25	0.7869674185	0.5585475494
10	10	0.25	0.786377709	0.557248243
10	10	0.75	0.7853457172	0.5560202126
10	1	1	0.7850508625	0.5564151599
6	1	0.25	0.7844611529	0.5520049028
2	1	0.5	0.7840188707	0.5547692441
2	1	0.75	0.7840188707	0.5553098304
2	10	1	0.7840188707	0.5533342045
2	1	1	0.7831343064	0.5522459451
2	5	1	0.7818074598	0.5486625425
6	10	0.25	0.7809228955	0.5448499169
10	10	1	0.7809228955	0.5469906803
2	1	0.25	0.7806280407	0.5471304348
2	10	0.75	0.7795960489	0.5440285826
2	10	0.5	0.7793011942	0.543816116
2	5	0.75	0.778121775	0.5413782595
2	5	0.5	0.7760577915	0.5372731625
2	10	0.25	0.773993808	0.5329270947
2	5	0.25	0.7729618163	0.5310596833

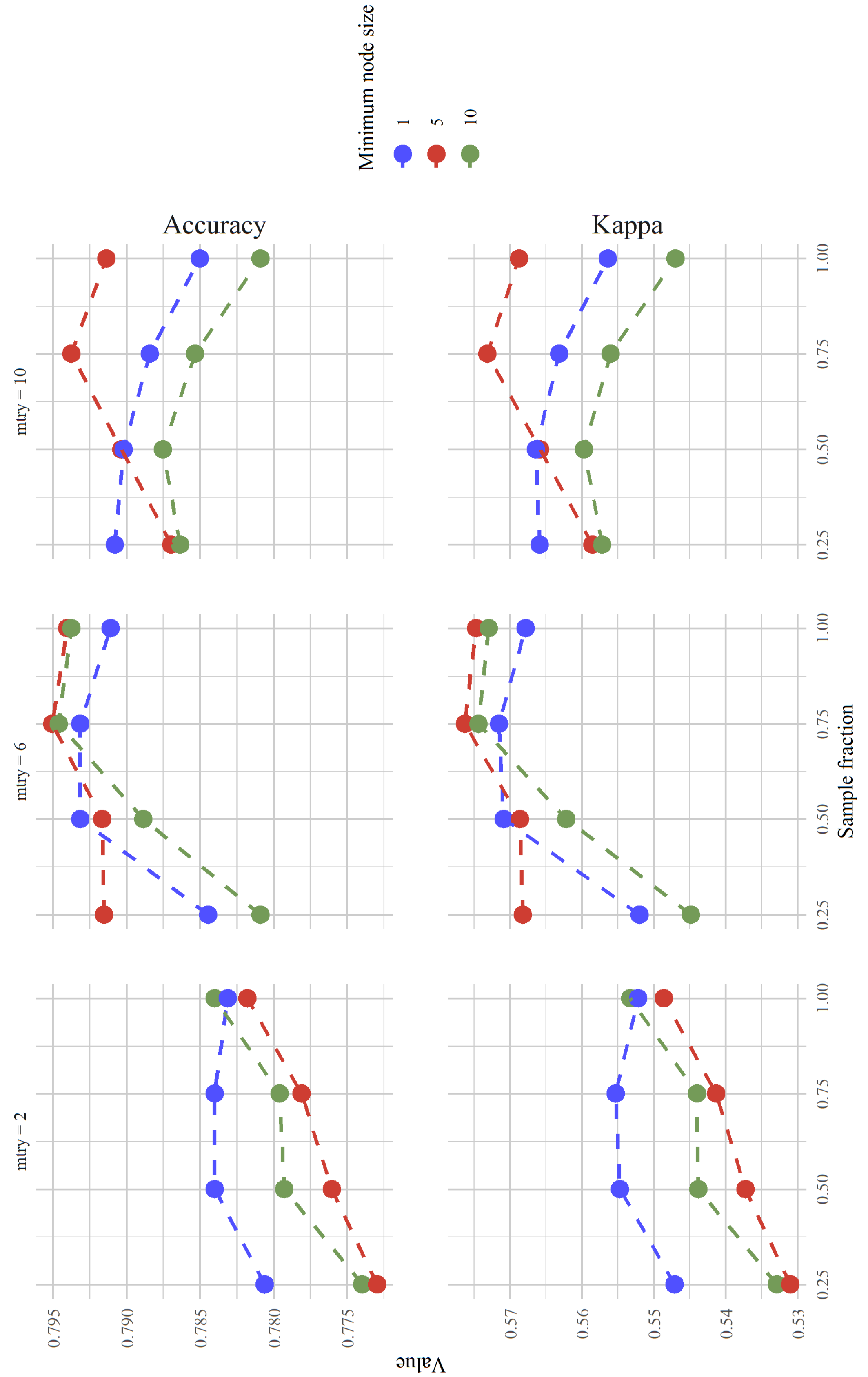
Minimum node size
1
5
10
Accuracy
Kappa
mtry = 2
mtry = 6
mtry = 10
Value
Sample fraction
0.25
0.50
0.75
1.00
0.795
0.790
0.785
0.780
0.775
0.57
0.56
0.55
0.54
0.53

Diagnosis step

mtry	min.node.size	sample.fraction	Accuracy	Kappa
10	1	1	0.8370045824	0.6591986937
10	1	0.75	0.8349947745	0.6545319018
6	1	1	0.8346732052	0.6541413899
6	1	0.75	0.8321006512	0.6484725345
10	1	0.5	0.8293673125	0.6424316812
6	5	1	0.8293673125	0.6429815814
10	5	1	0.8273575046	0.6394348968
10	10	1	0.8267947584	0.6386236477
6	1	0.5	0.8247849506	0.632820303
6	5	0.75	0.8245839698	0.6327835746
10	5	0.75	0.8232976927	0.6308984047
10	10	0.75	0.8222525927	0.6289912743
6	5	0.5	0.8187957231	0.6202190396
6	10	1	0.8187957231	0.6220657277
10	1	0.25	0.8154996382	0.6127609314
10	5	0.5	0.8154996382	0.6144060677
6	10	0.75	0.8140927727	0.6122615184
10	10	0.5	0.8140123804	0.6116194016
6	1	0.25	0.8136908112	0.609298797
6	10	0.5	0.8040839296	0.5914305559
6	5	0.25	0.7996221561	0.5800488609
10	5	0.25	0.7984966637	0.5783902632
10	10	0.25	0.7939544979	0.569677636
6	10	0.25	0.7882466436	0.5579371359
2	1	1	0.7821770239	0.5403564894
2	1	0.75	0.7808103545	0.5383564129
2	5	1	0.7802878045	0.537708721
2	1	0.5	0.7794436852	0.5352756839
2	5	0.75	0.778880939	0.5345629918
2	10	1	0.777594662	0.5310733095
2	5	0.5	0.7772328965	0.5316770486
2	10	0.75	0.7754240695	0.5259572611
2	1	0.25	0.7730123	0.5220366855
2	10	0.5	0.7720475923	0.5192146984
2	5	0.25	0.7702387652	0.517113203
2	10	0.25	0.7671436611	0.5092282184

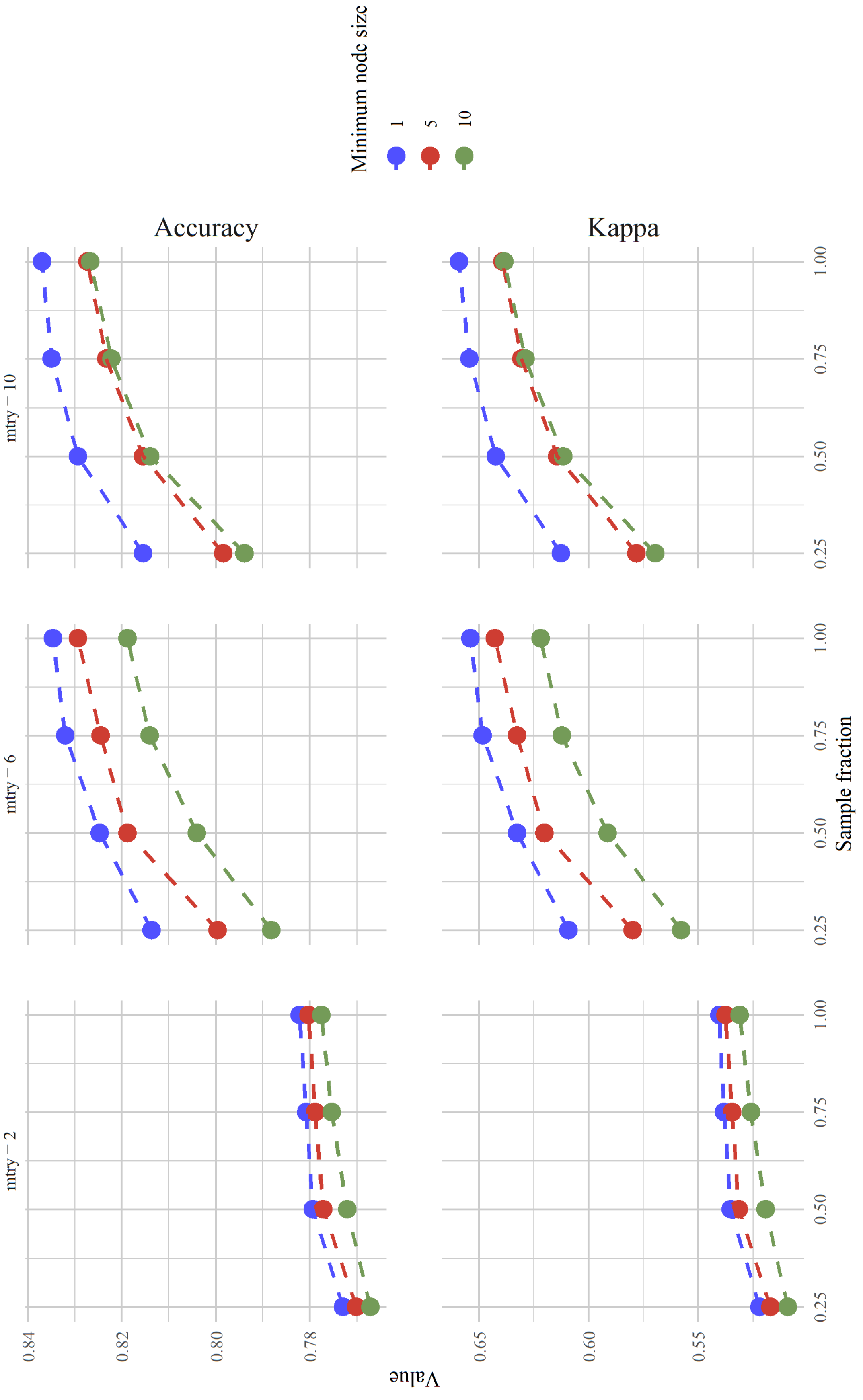
Minimum node size
1
5
10
Accuracy
Kappa
mtry = 2
mtry = 6
mtry = 10
Value
Sample fraction
0.84
0.82
0.80
0.78
0.65
0.60
0.55
0.25
0.50
0.75
1.00

Treatment step

mtry	min.node.size	sample.fraction	Accuracy	Kappa
10	1	1	0.8842497154	0.7585941367
10	1	0.75	0.8817574844	0.7531995706
10	5	1	0.8799421556	0.7498168096
6	5	1	0.8787421925	0.7471054682
6	1	1	0.8784960463	0.7465828146
6	1	0.75	0.8757269007	0.740659885
10	5	0.75	0.8751423033	0.7398336753
10	1	0.5	0.8746192425	0.7380311699
6	5	0.75	0.8735115843	0.7362062169
6	1	0.5	0.8710193533	0.7305219851
10	10	1	0.8706809021	0.7309784845
10	5	0.5	0.8665579521	0.7218215971
10	10	0.75	0.8656349035	0.7205017784
6	5	0.5	0.8652041476	0.7188757597
6	10	1	0.865142611	0.7193597073
10	1	0.25	0.8616657949	0.7106767913
6	1	0.25	0.8597889296	0.7069641824
6	10	0.75	0.8590504908	0.7066980088
10	10	0.5	0.8566813329	0.7022446259
10	5	0.25	0.8504353712	0.6890438374
6	5	0.25	0.8478816036	0.6834653447
6	10	0.5	0.8462508846	0.6806766416
10	10	0.25	0.8374203871	0.6630715977
6	10	0.25	0.8303129134	0.6487612705
2	1	0.75	0.8232977447	0.6336604791
2	1	1	0.8232362081	0.6337398337
2	5	1	0.8219131719	0.6302070023
2	5	0.75	0.8205901357	0.6276670468
2	1	0.5	0.8204978308	0.6280624419
2	10	1	0.8190209532	0.6244276202
2	5	0.5	0.8174517707	0.6210571457
2	10	0.75	0.8160671979	0.6183814544
2	1	0.25	0.8152979908	0.6172288465
2	10	0.5	0.812713455	0.6116958745
2	5	0.25	0.8098520046	0.6060252814
2	10	0.25	0.8060367373	0.5979738348

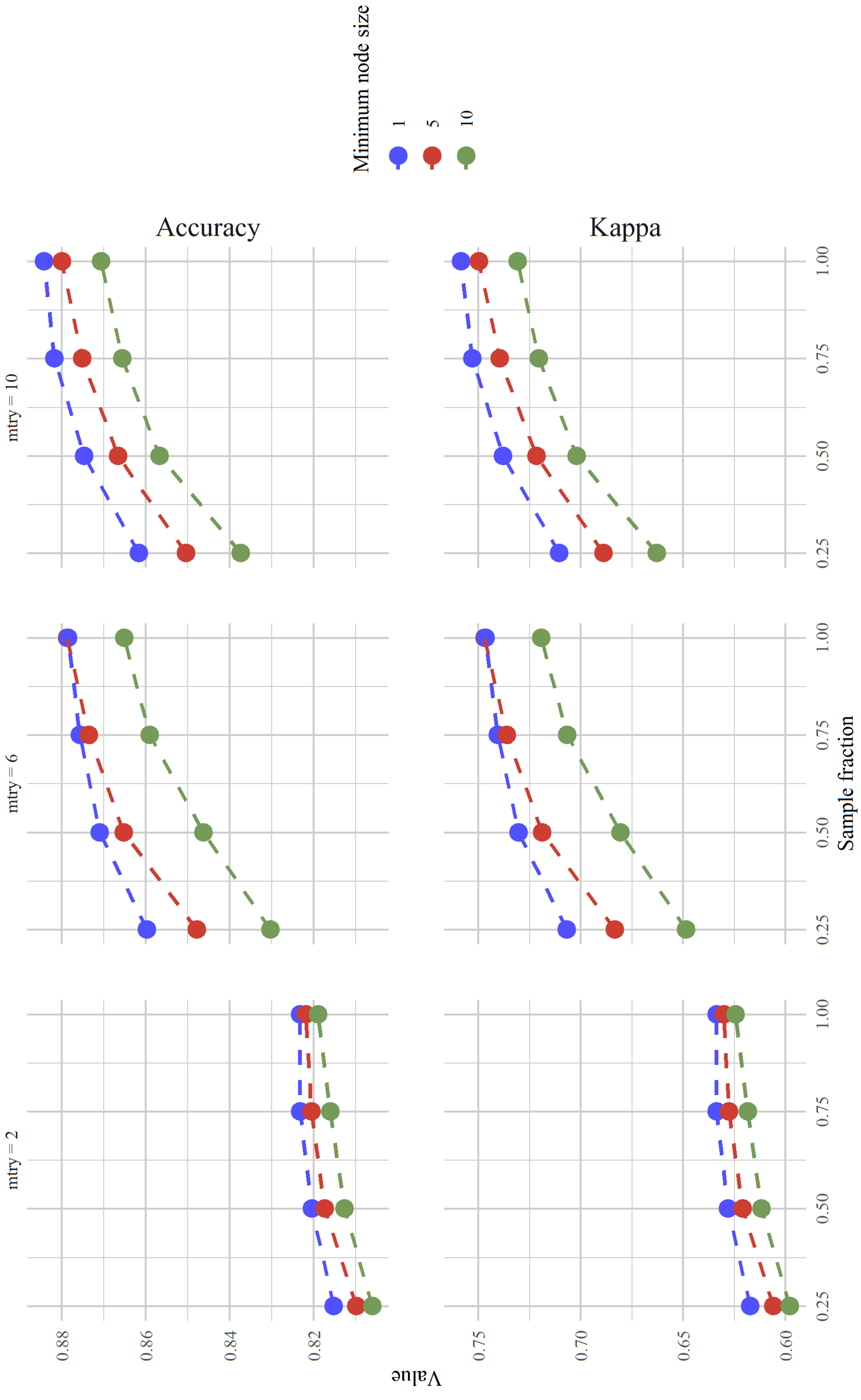

Accuracy
Kappa
mtry = 2
mtry = 6
mtry = 10
Minimum node size
1
5
10
Value
Sample fraction
0.25
0.50
0.75
1.00
0.88
0.86
0.84
0.82
0.75
0.70
0.65
0.60

Control step

mtry	min.node.size	sample.fraction	Accuracy	Kappa
10	5	0.25	0.7580799388	0.5002539647
6	1	0.5	0.7573149742	0.4966853646
6	10	1	0.7567412507	0.4982531275
6	1	0.25	0.7552113215	0.4920058964
6	1	0.75	0.7550200803	0.4930747922
10	5	0.5	0.7548288392	0.49333785
6	1	1	0.7542551157	0.4919514262
6	10	0.75	0.7532989099	0.4913249211
6	5	0.75	0.7531076688	0.4883655098
6	5	1	0.7531076688	0.4885971366
6	5	0.5	0.7529164276	0.4867196368
2	5	1	0.7513864984	0.4821306624
6	5	0.25	0.7511952572	0.4838764523
10	5	0.75	0.7511952572	0.4864956301
10	5	1	0.7506215338	0.4861517676
2	5	0.5	0.749665328	0.4797592687
2	5	0.75	0.749665328	0.4787530576
10	1	0.25	0.7494740868	0.4801587302
6	10	0.5	0.7494740868	0.4833802817
10	10	1	0.7479441576	0.4796390299
10	1	0.5	0.7477529164	0.4780962071
10	10	0.25	0.7475616753	0.4782019426
10	10	0.5	0.7475616753	0.4784375706
2	1	1	0.7469879518	0.4737769191
10	10	0.75	0.7458405049	0.4749703708
2	5	0.25	0.7429718876	0.4649072915
6	10	0.25	0.7429718876	0.4697328373
2	1	0.5	0.7408682348	0.4599442009
2	1	0.75	0.7402945114	0.4579769643
10	1	0.75	0.7389558233	0.4614170565
2	10	0.75	0.7383820998	0.4544211486
2	10	1	0.7383820998	0.4546697039
2	1	0.25	0.7368521706	0.4511055391
10	1	1	0.736087206	0.4557746479
2	10	0.5	0.734939759	0.4482484076
2	10	0.25	0.7303499713	0.4399046646

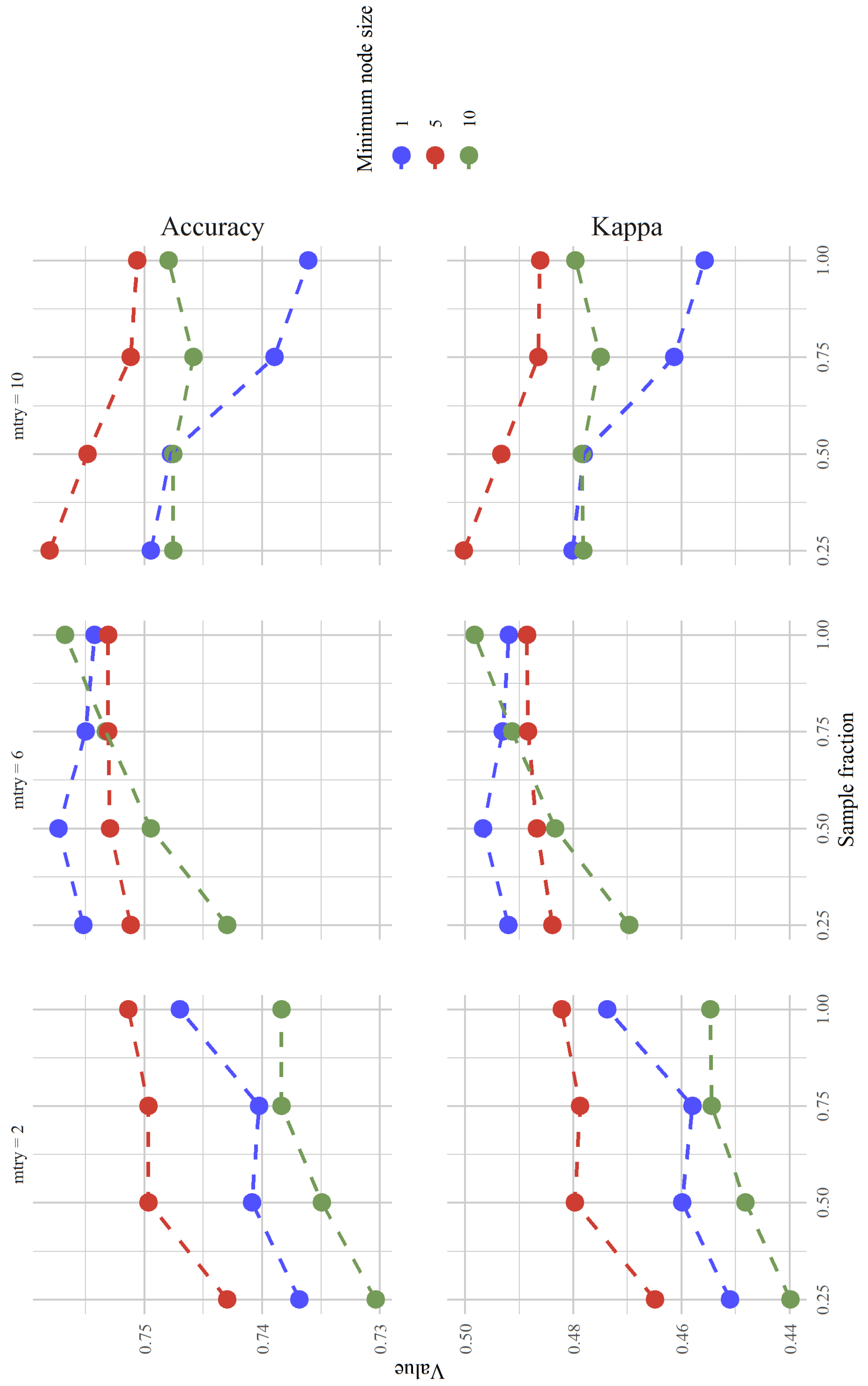
Minimum node size
1
5
10
Accuracy
Kappa
mtry = 2
mtry = 6
mtry = 10
Value
Sample fraction
0.25
0.50
0.75
1.00
0.73
0.74
0.75
0.44
0.46
0.48
0.50

Supplemental Figures

Supplemental figure S1 – Interactions between age, sex, and area of residence for screening

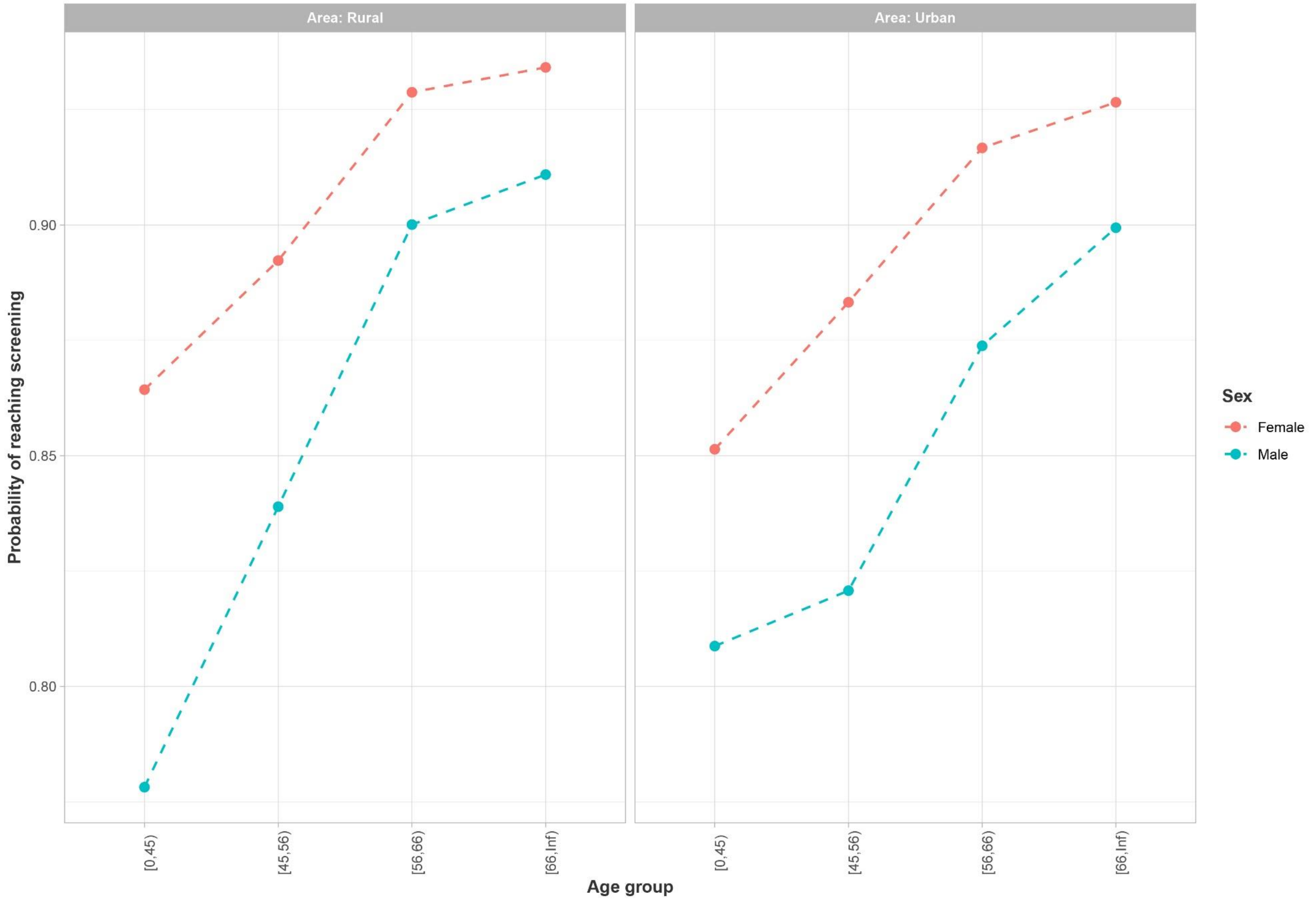

Supplemental figure S2 – Interactions between age, sex, and area of residence for diagnosis

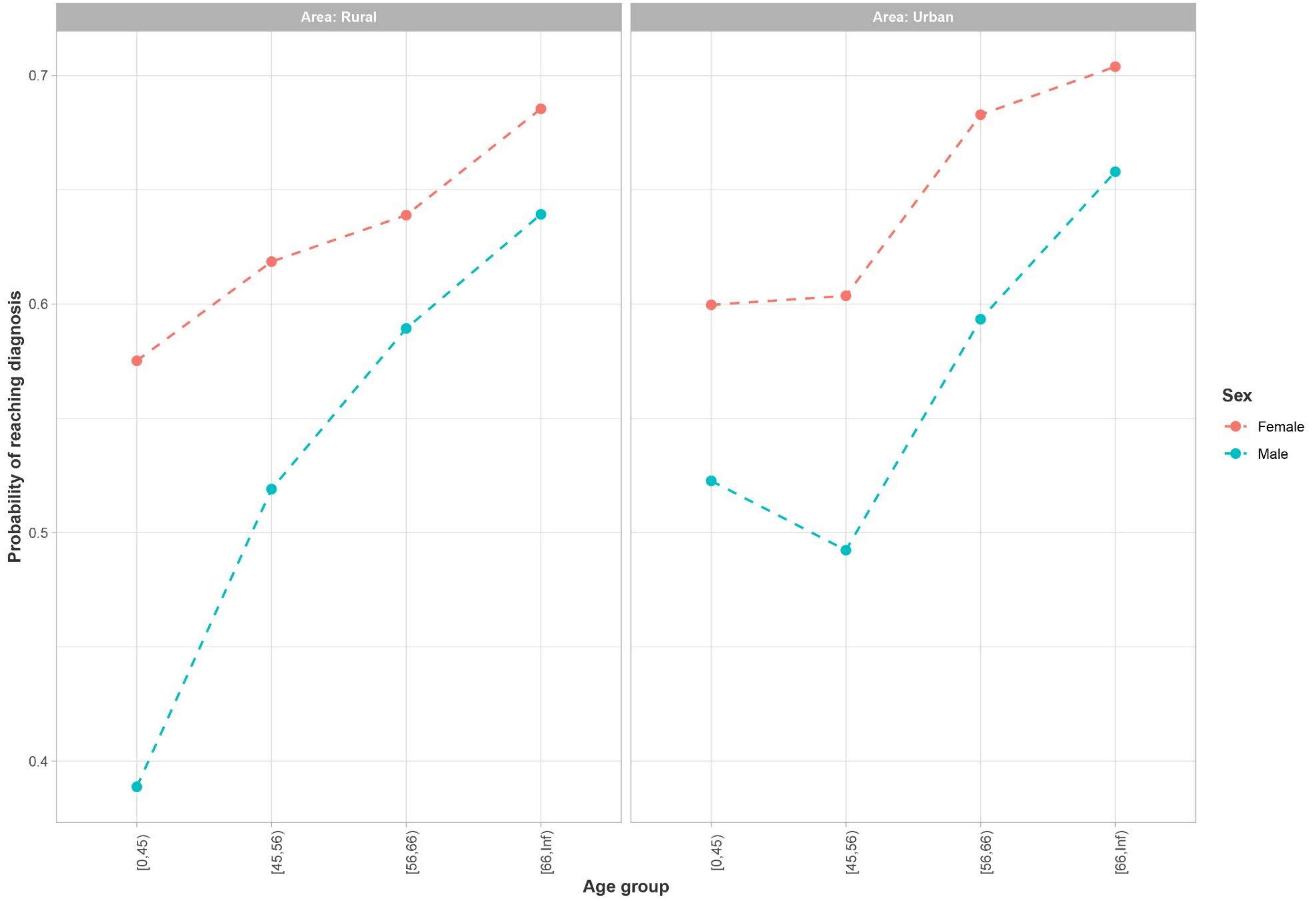

Supplemental figure S3 – Interactions between age, sex, and area of residence for treatment

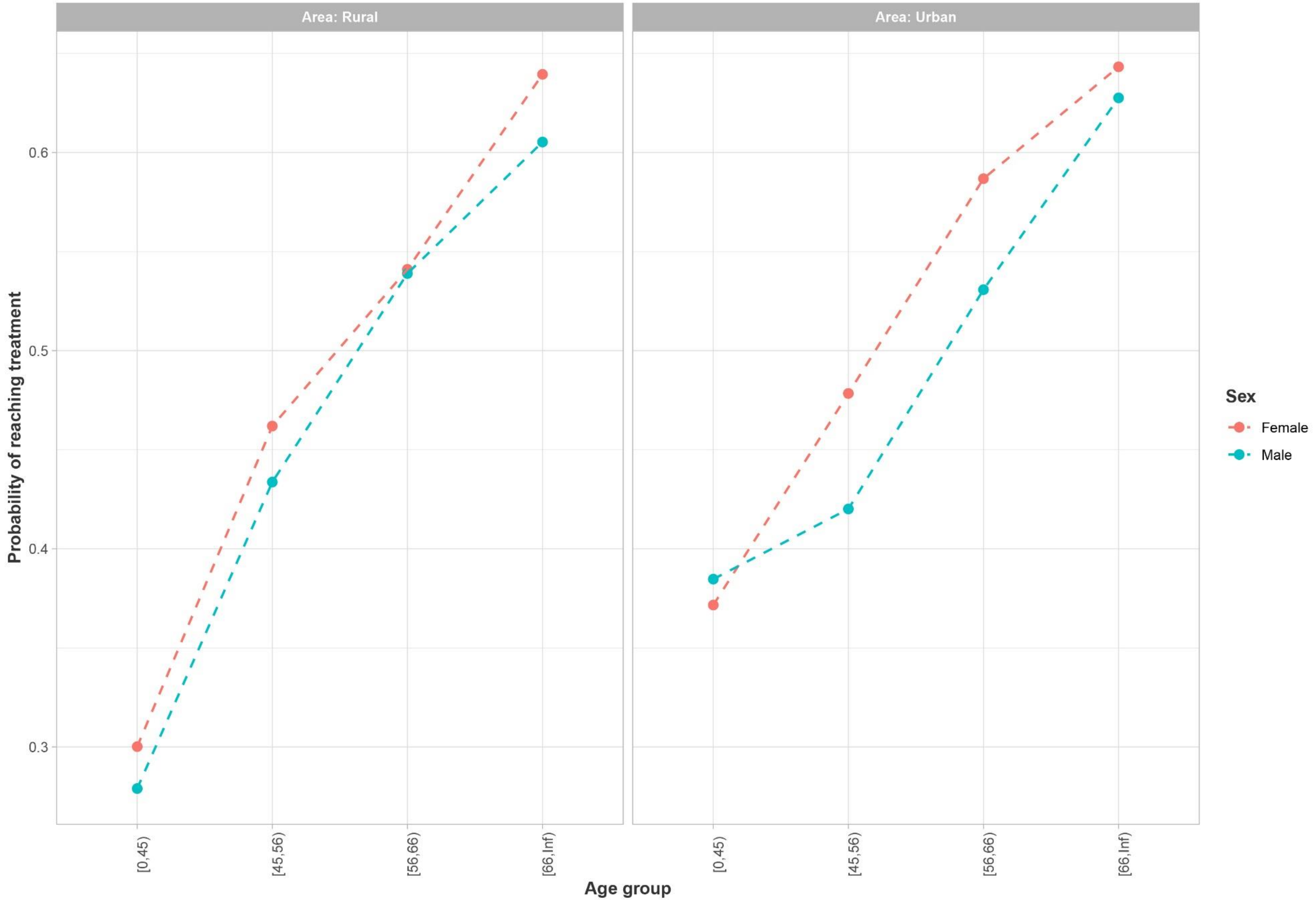

Supplemental figure S4 – Interactions between age, sex, and area of residence for control

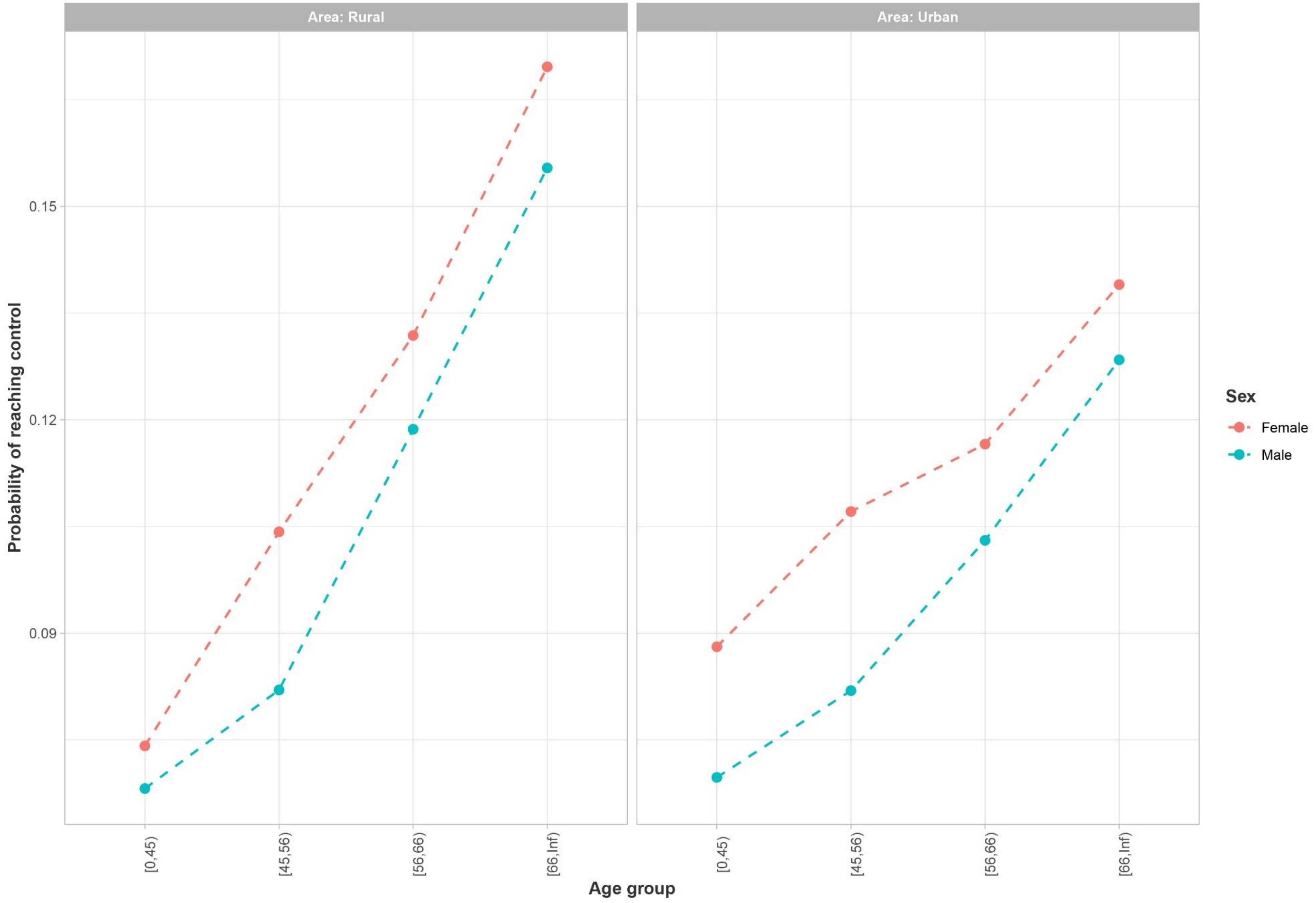

Supplemental figure S5 – Interactions between aera of residence, education, and wealth index for screening

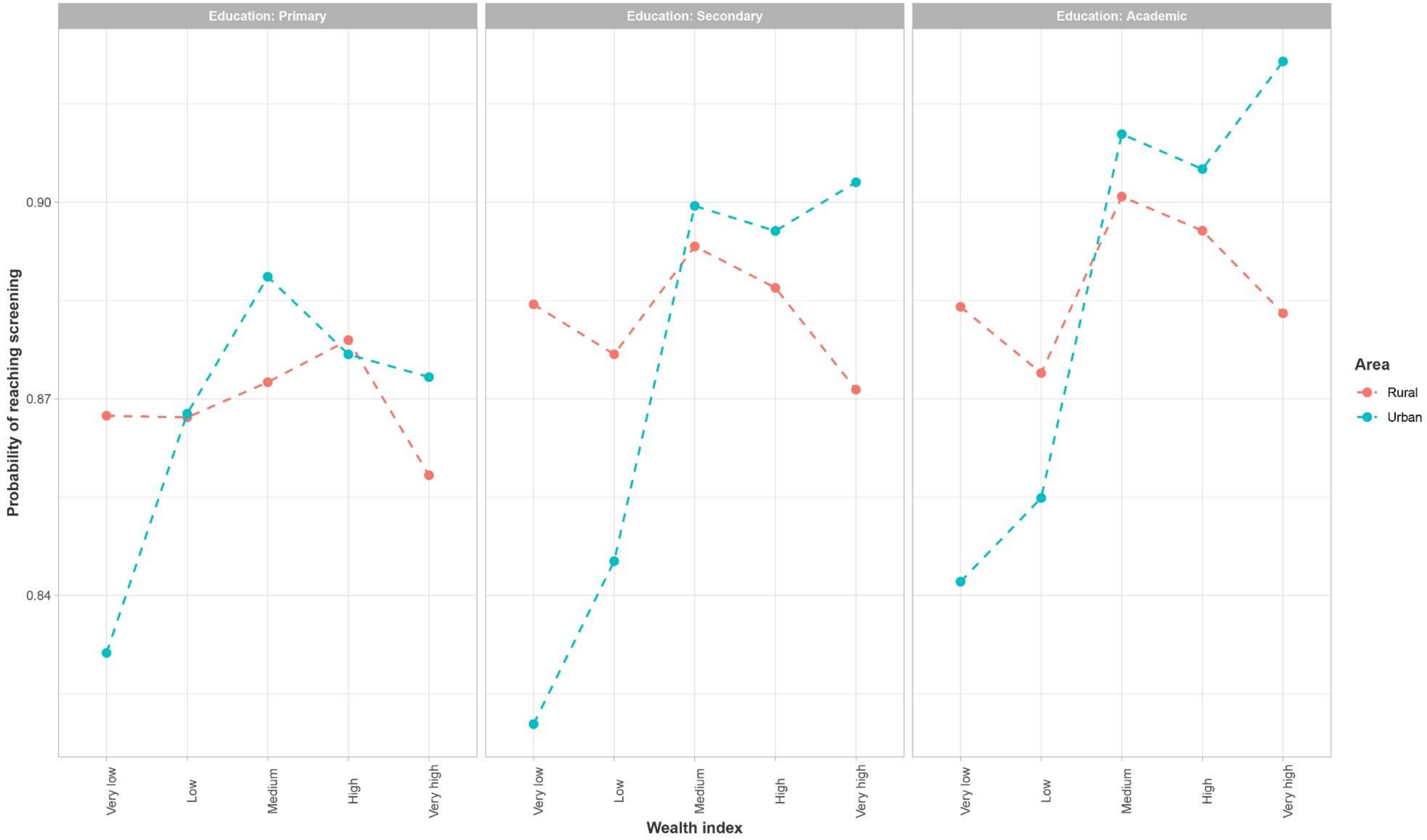

Supplemental figure S6 – Interactions between aera of residence, education, and wealth index for diagnosis

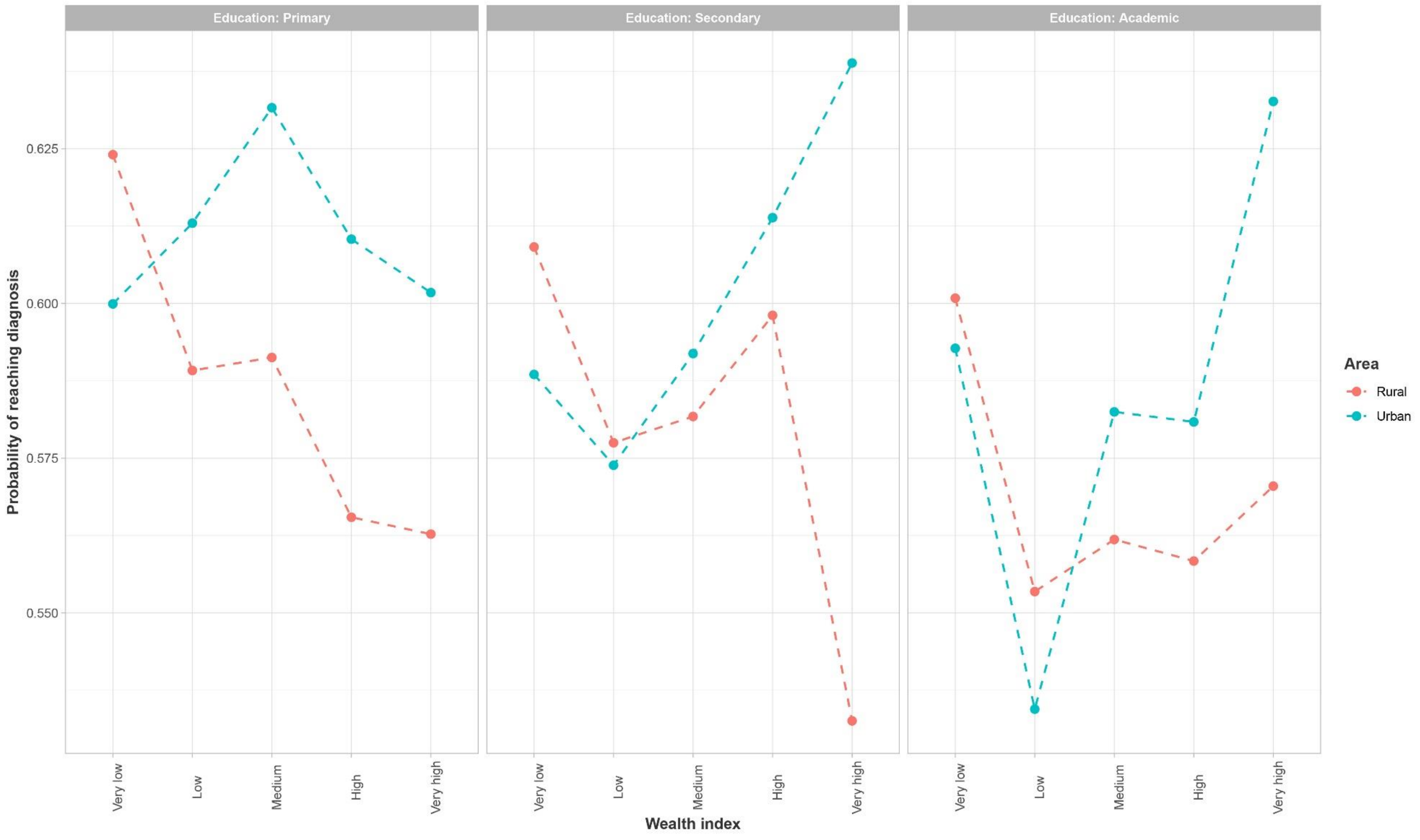

Supplemental figure S7 – Interactions between aera of residence, education, and wealth index for treatment

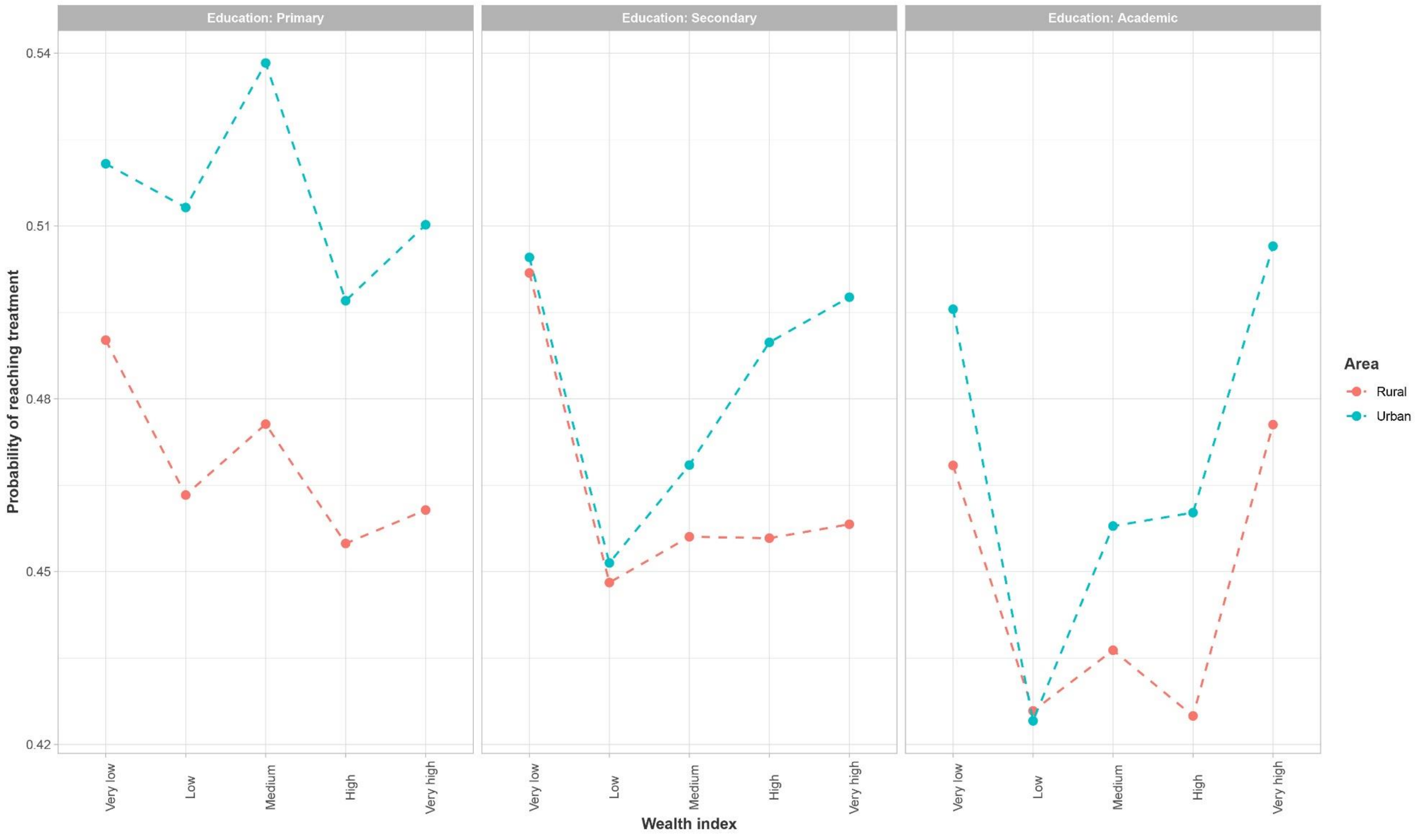

Supplemental figure S8 – Interactions between aera of residence, education, and wealth index for control

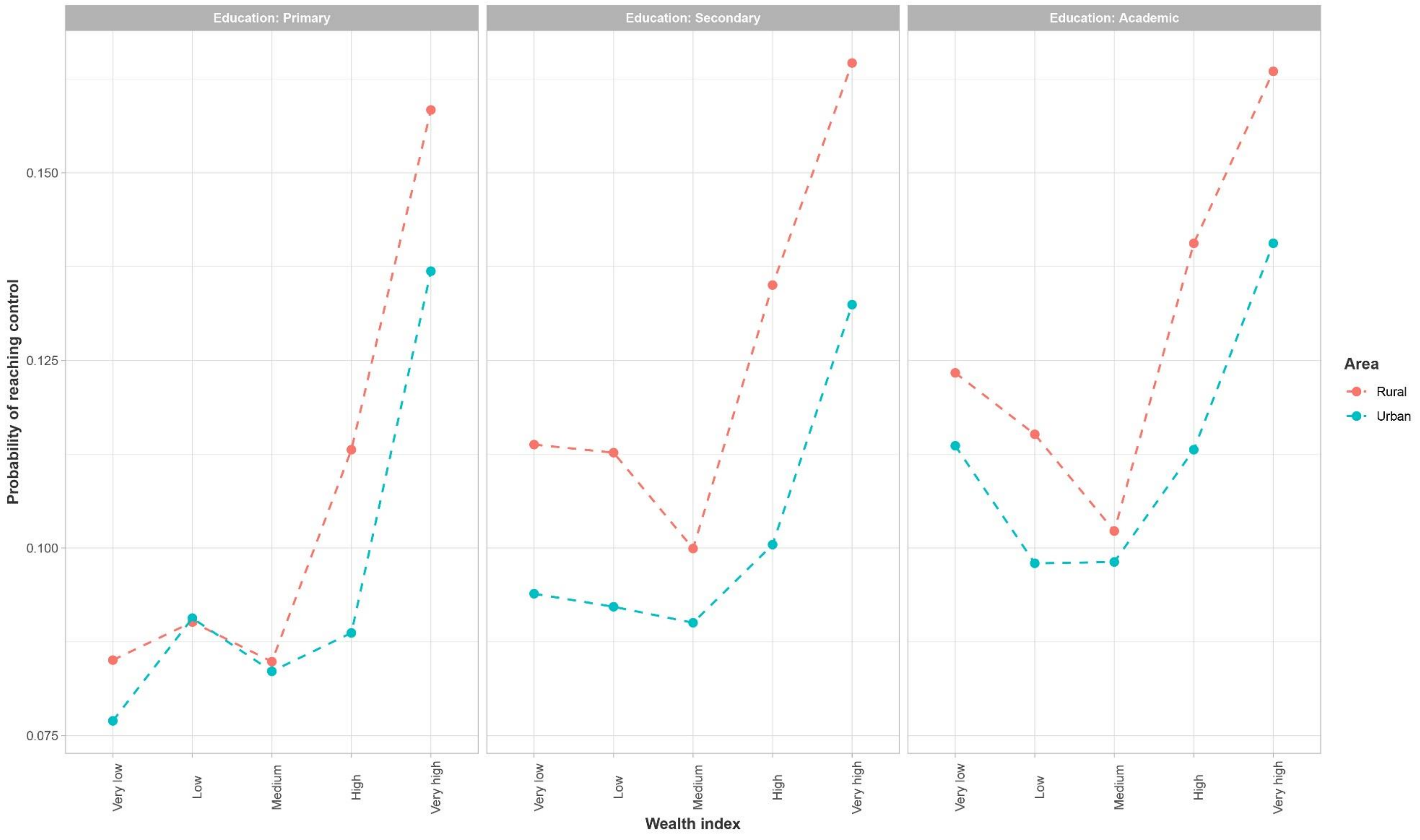

Supplemental figure S9 – Interactions between age, sex, education, and wealth index for screening

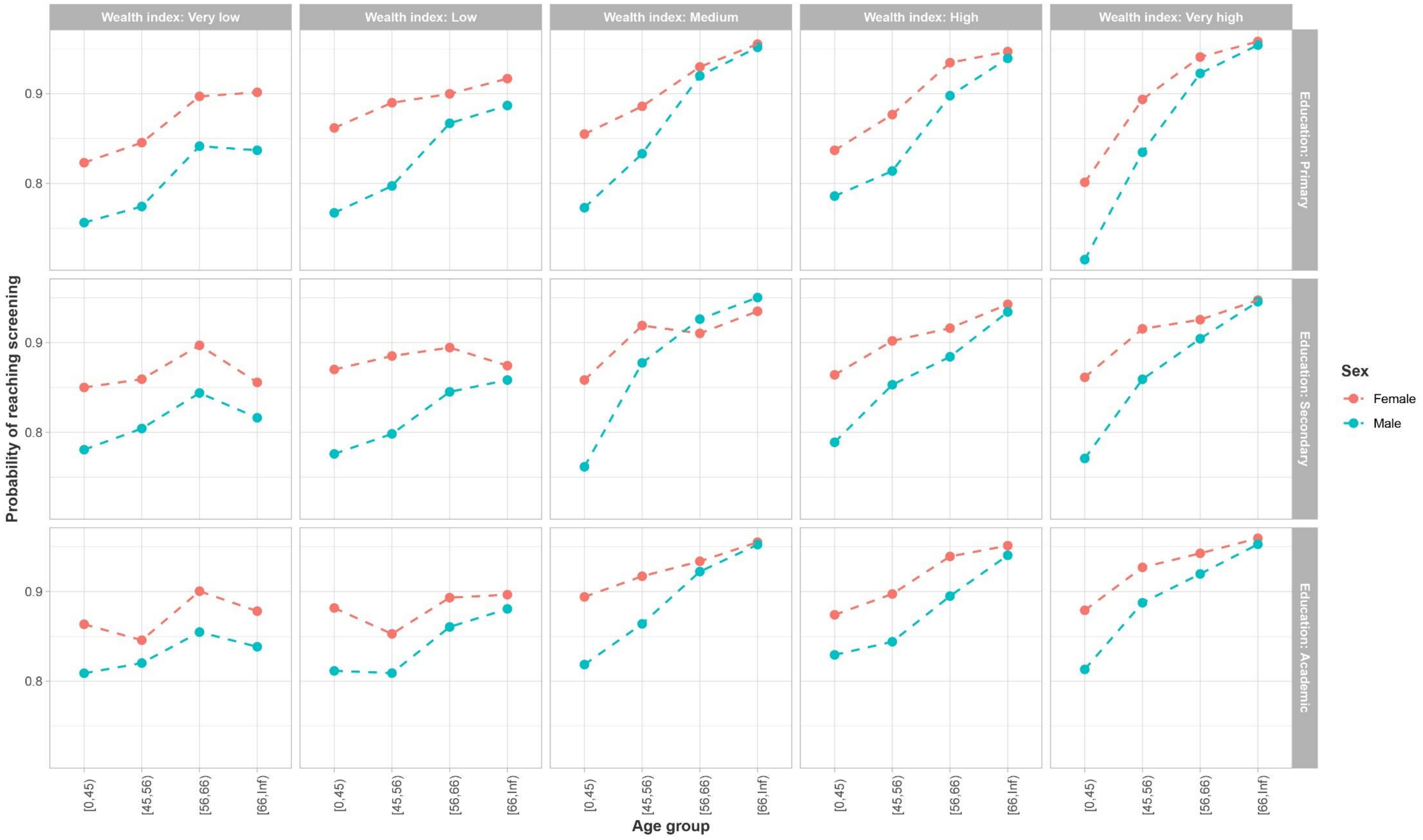

Supplemental figure S10 – Interactions between age, sex, education, and wealth index for diagnosis

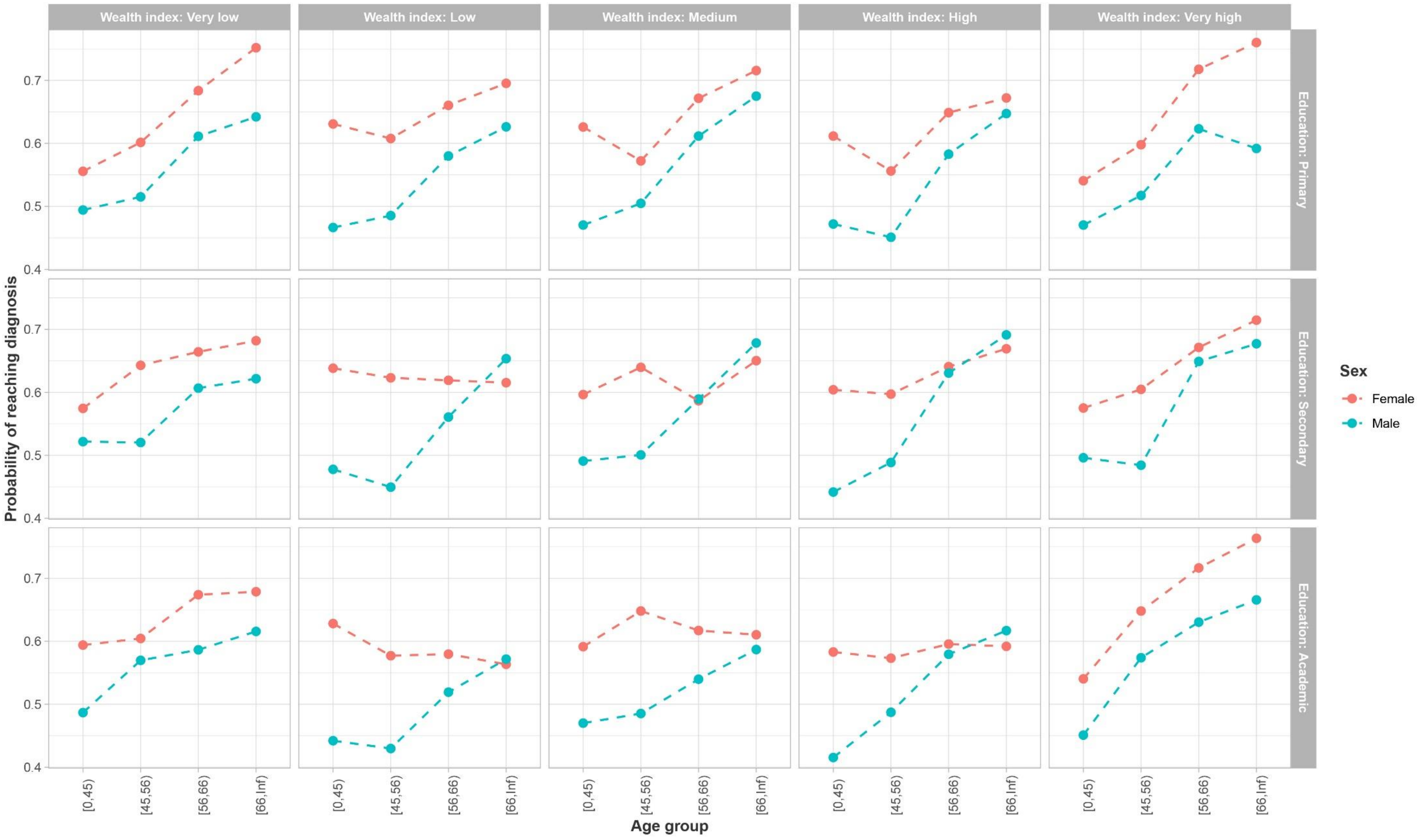

Supplemental figure S11 – Interactions between age, sex, education, and wealth index for treatment

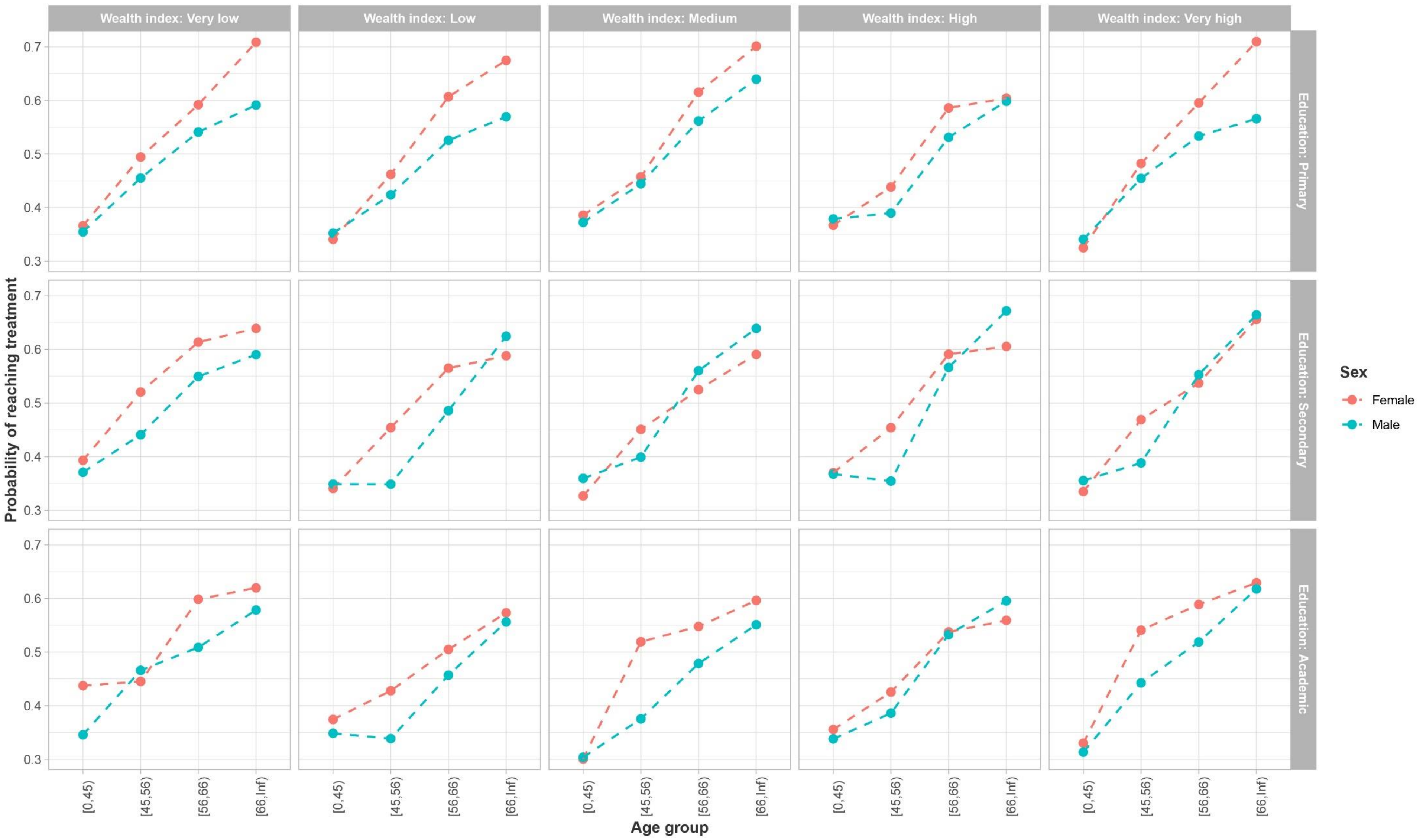

Supplemental figure S12 – Interactions between age, sex, education, and wealth index for control

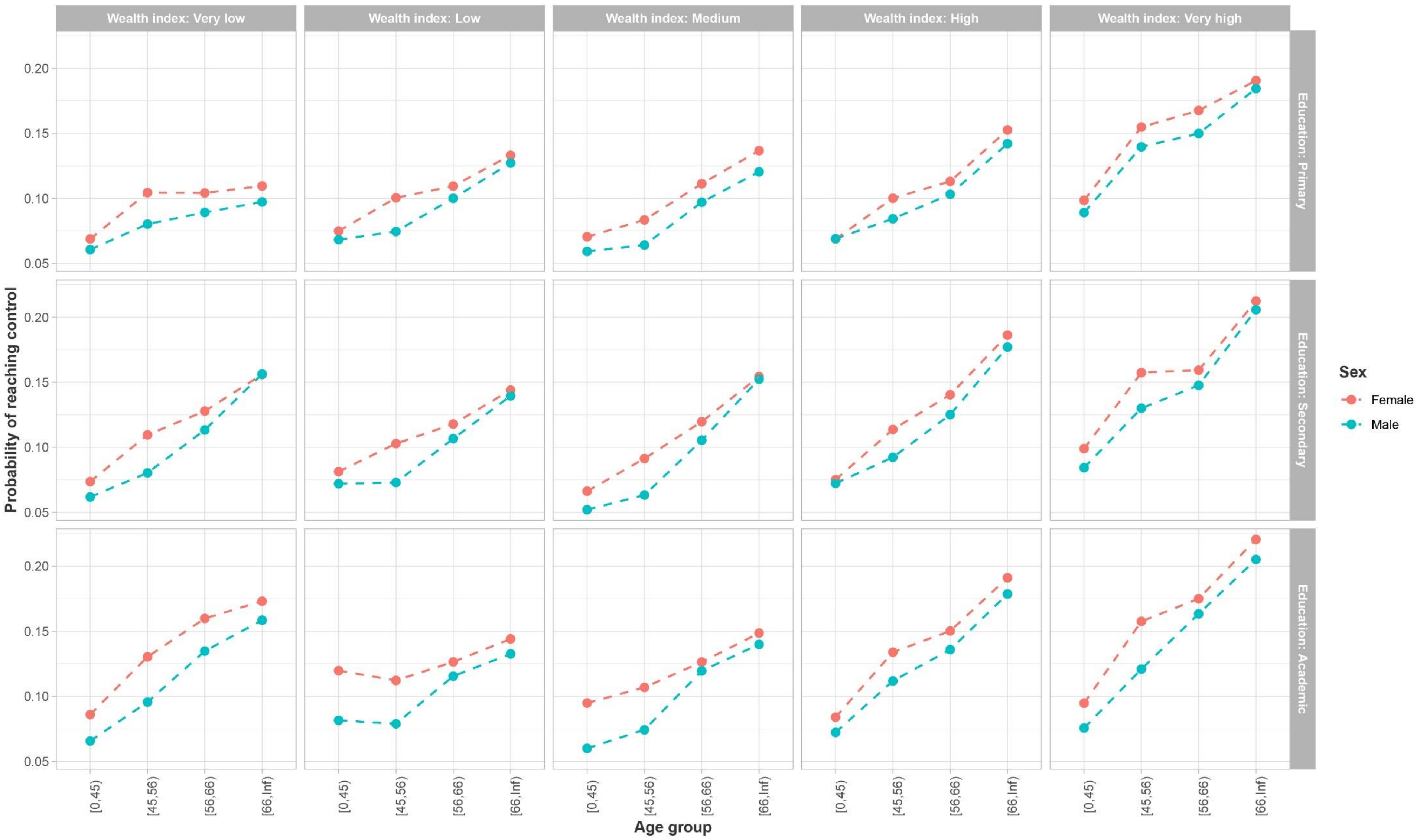

Supplemental figure S13 – Interactions between age, sex, marital status, and wealth index for screening

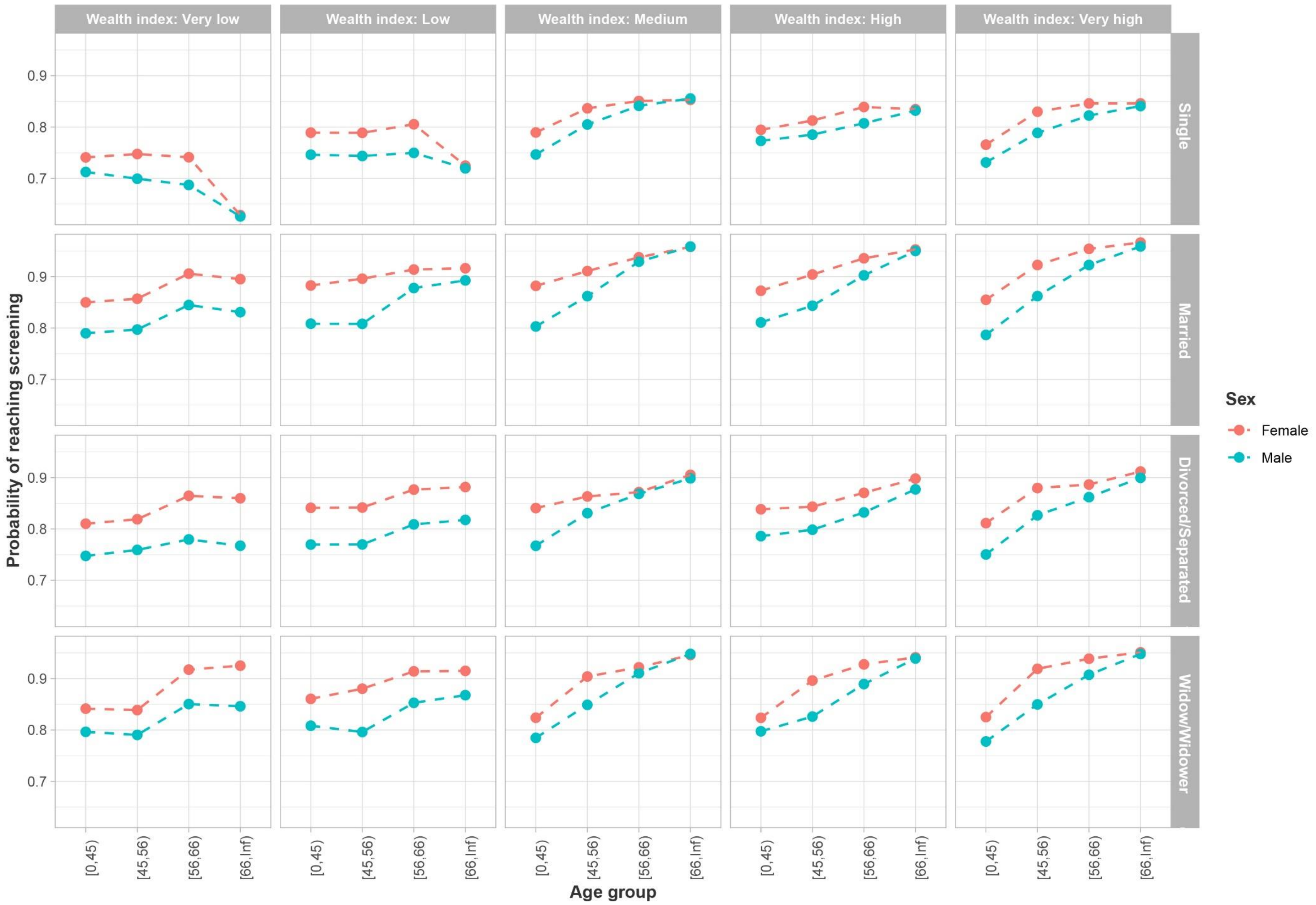

Supplemental figure S14 – Interactions between age, sex, marital status, and wealth index for diagnosis

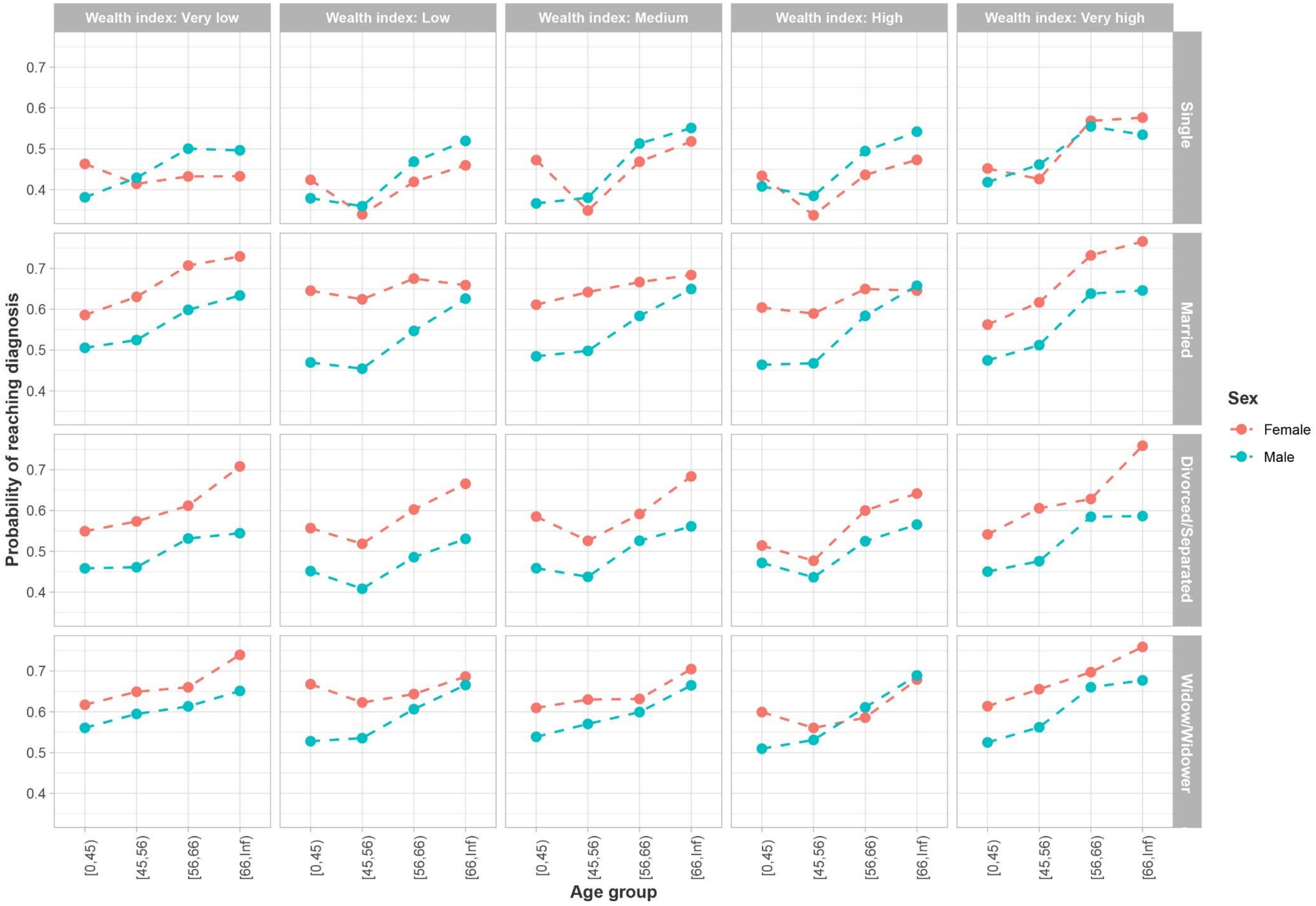

Supplemental figure S15 – Interactions between age, sex, marital status, and wealth index for treatment

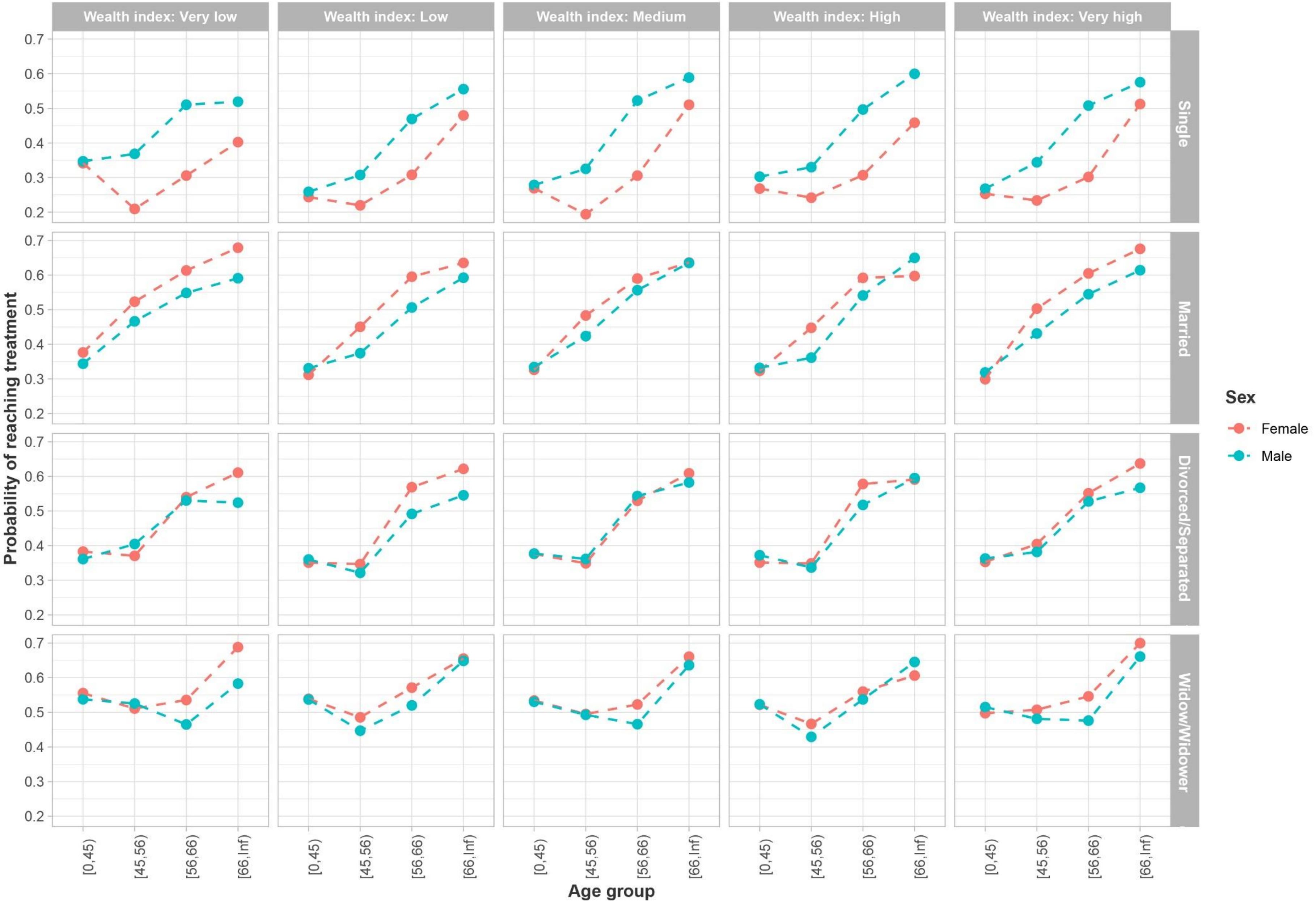

Supplemental figure S16 – Interactions between age, sex, marital status, and wealth index for control

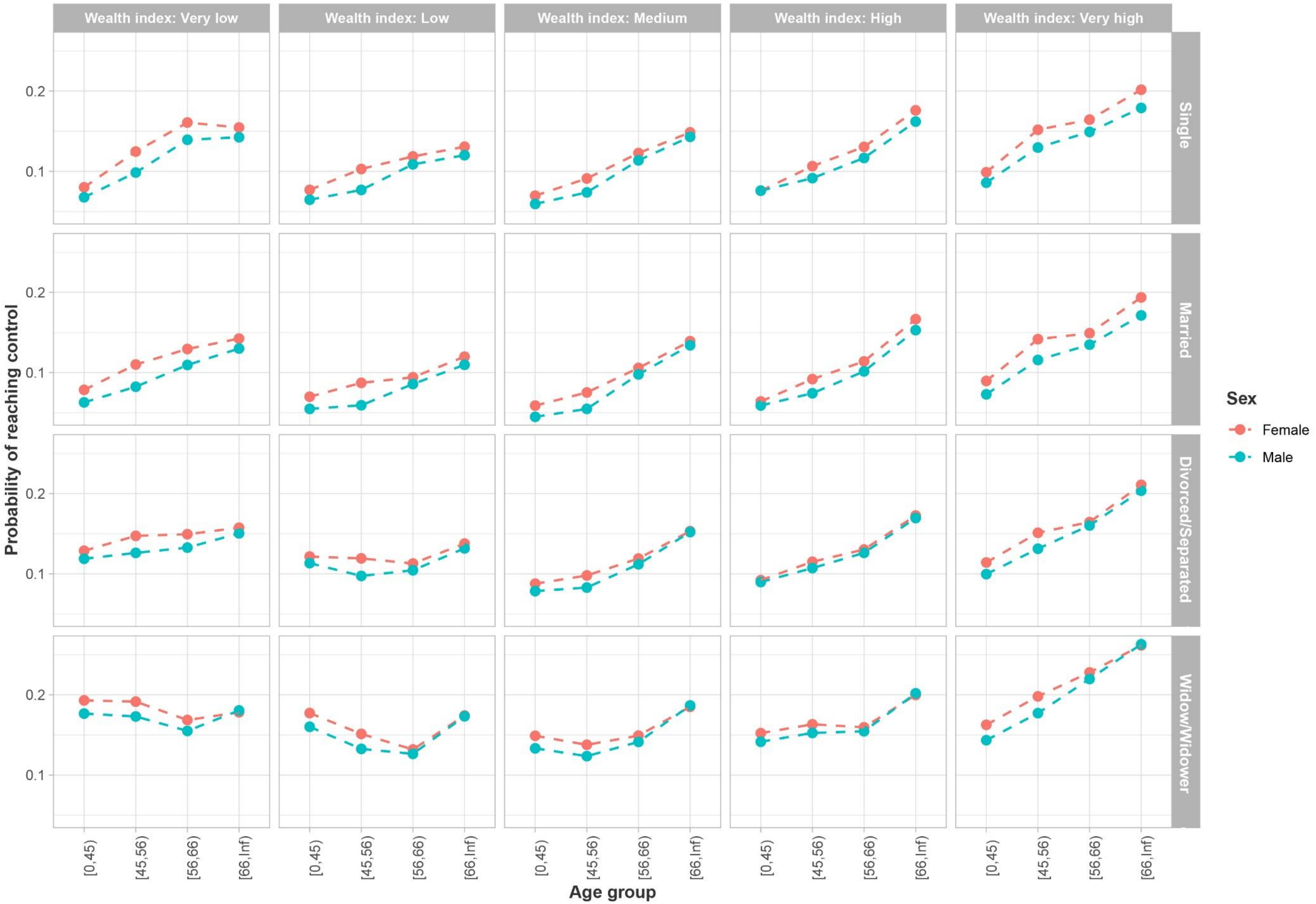

Supplemental figure S17 – Interactions between age, sex, area of residence, and marital status for screening

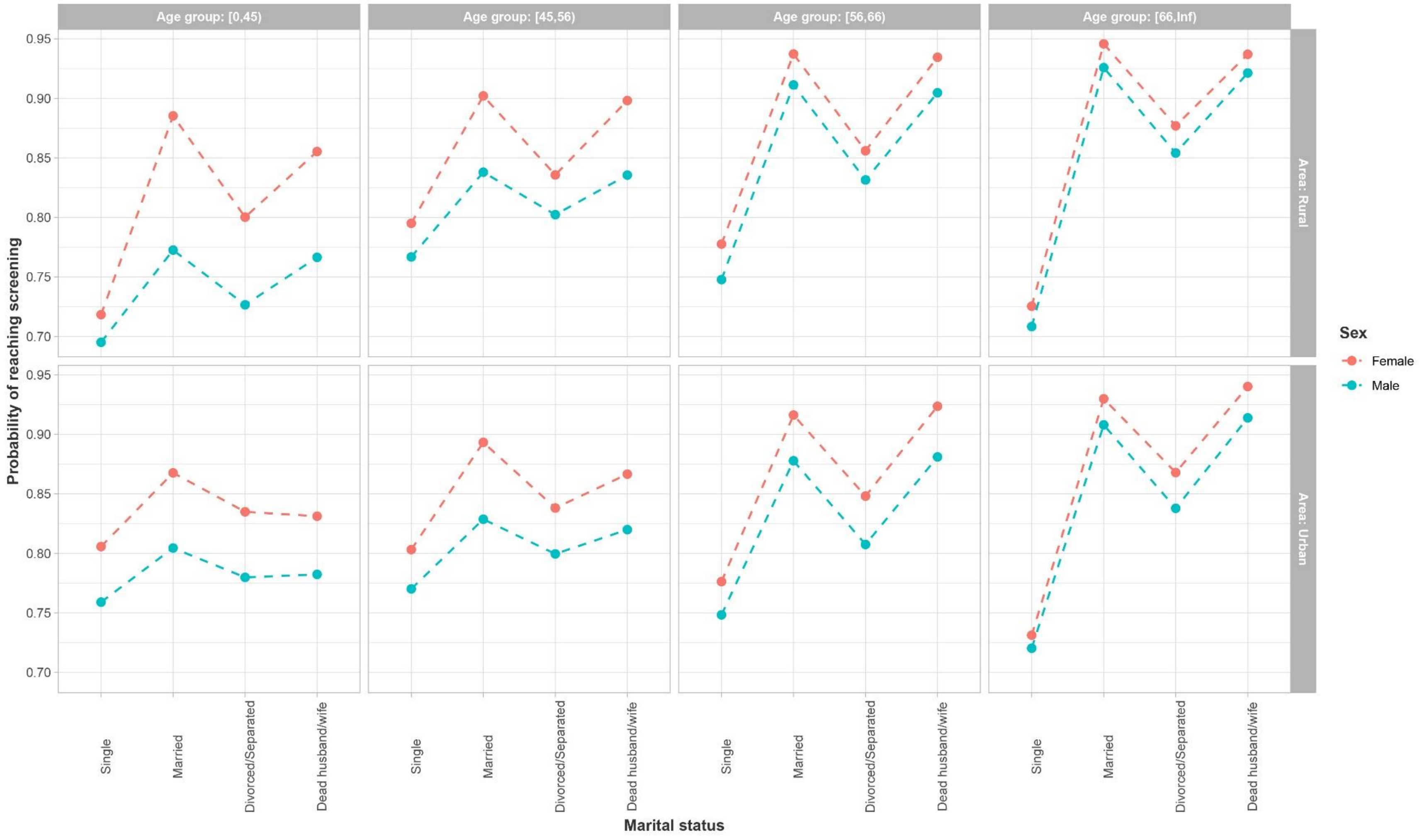

Supplemental figure S18 – Interactions between age, sex, area of residence, and marital status for diagnosis

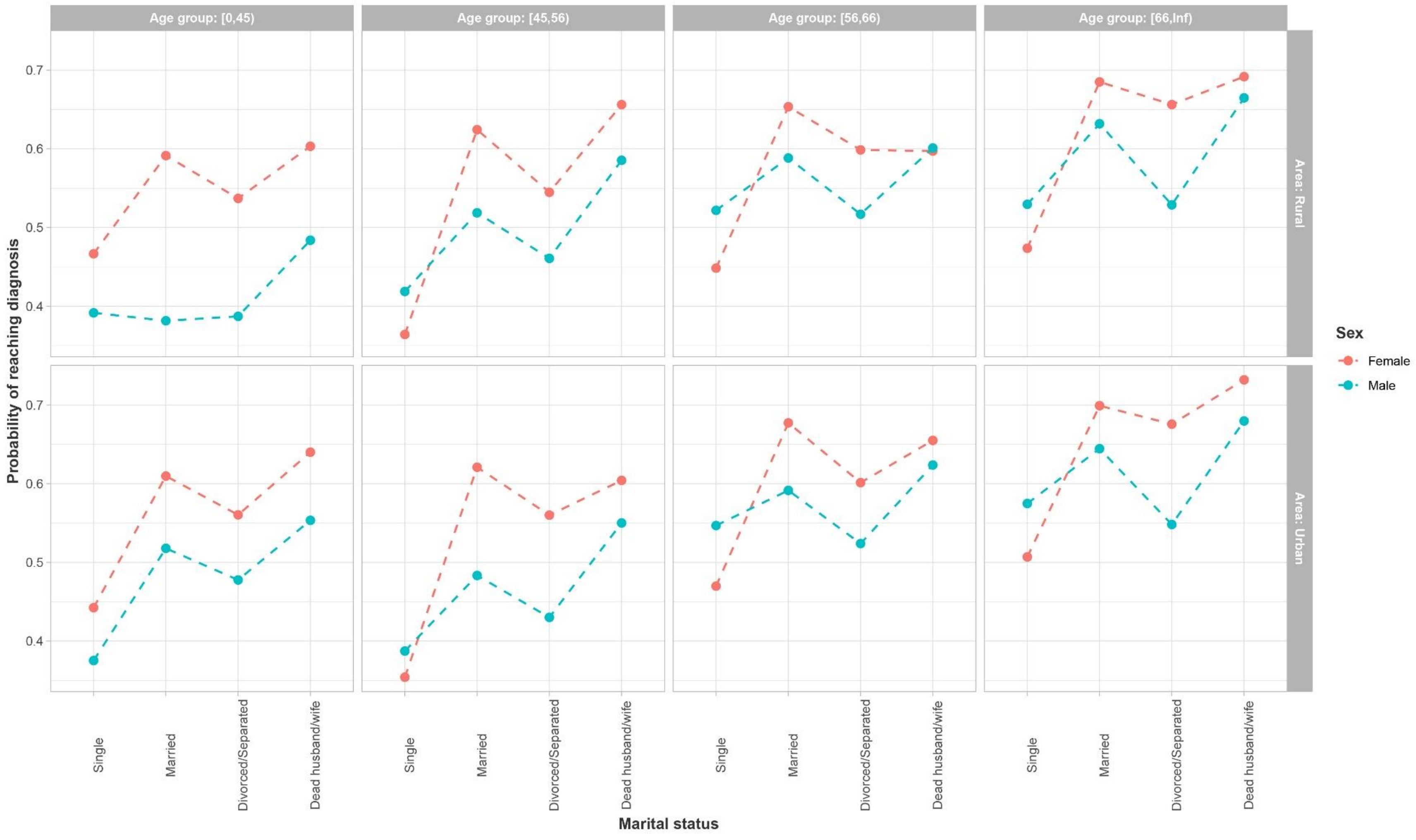

Supplemental figure S19 – Interactions between age, sex, area of residence, and marital status for treatment

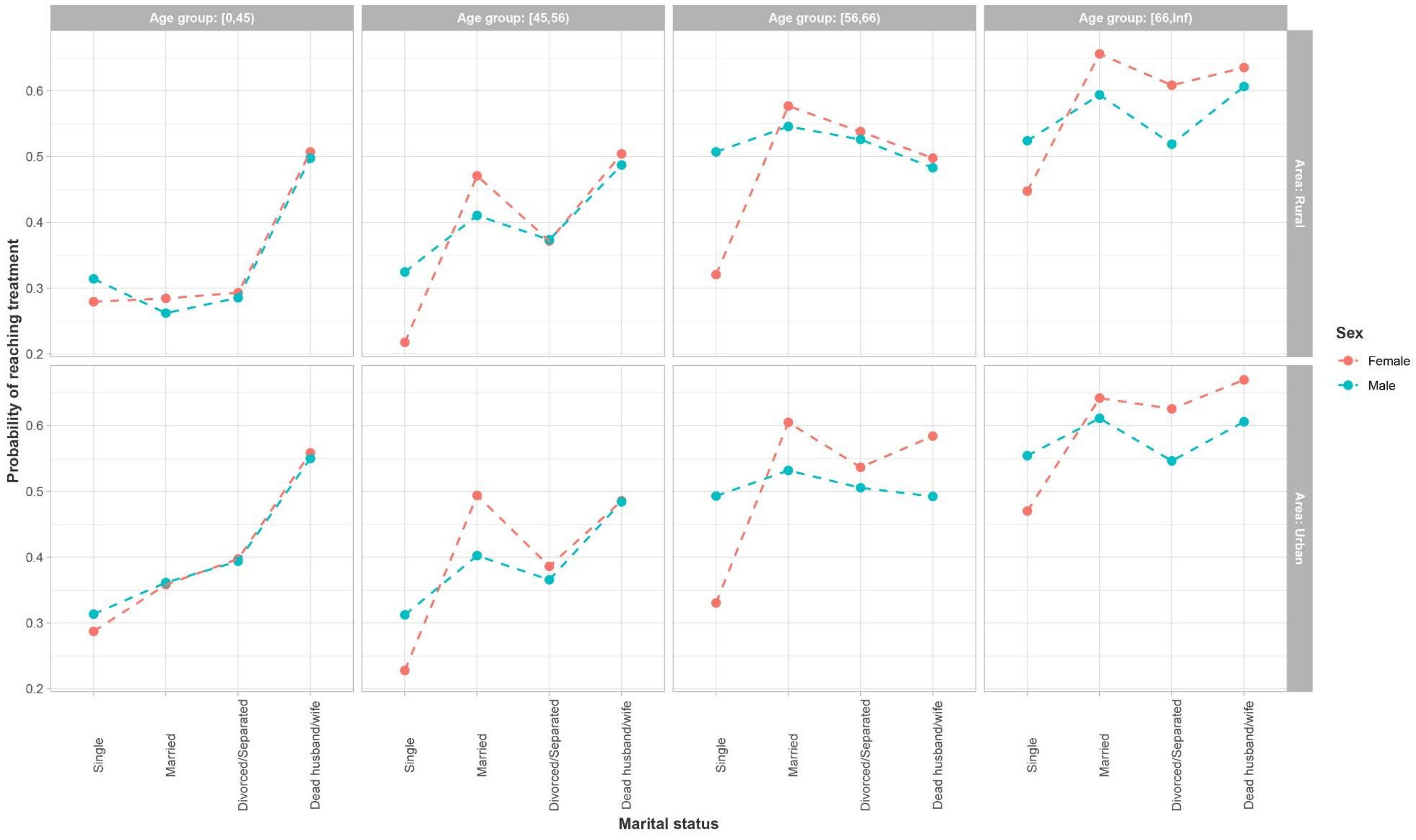

Supplemental figure S20 – Interactions between age, sex, area of residence, and marital status for control

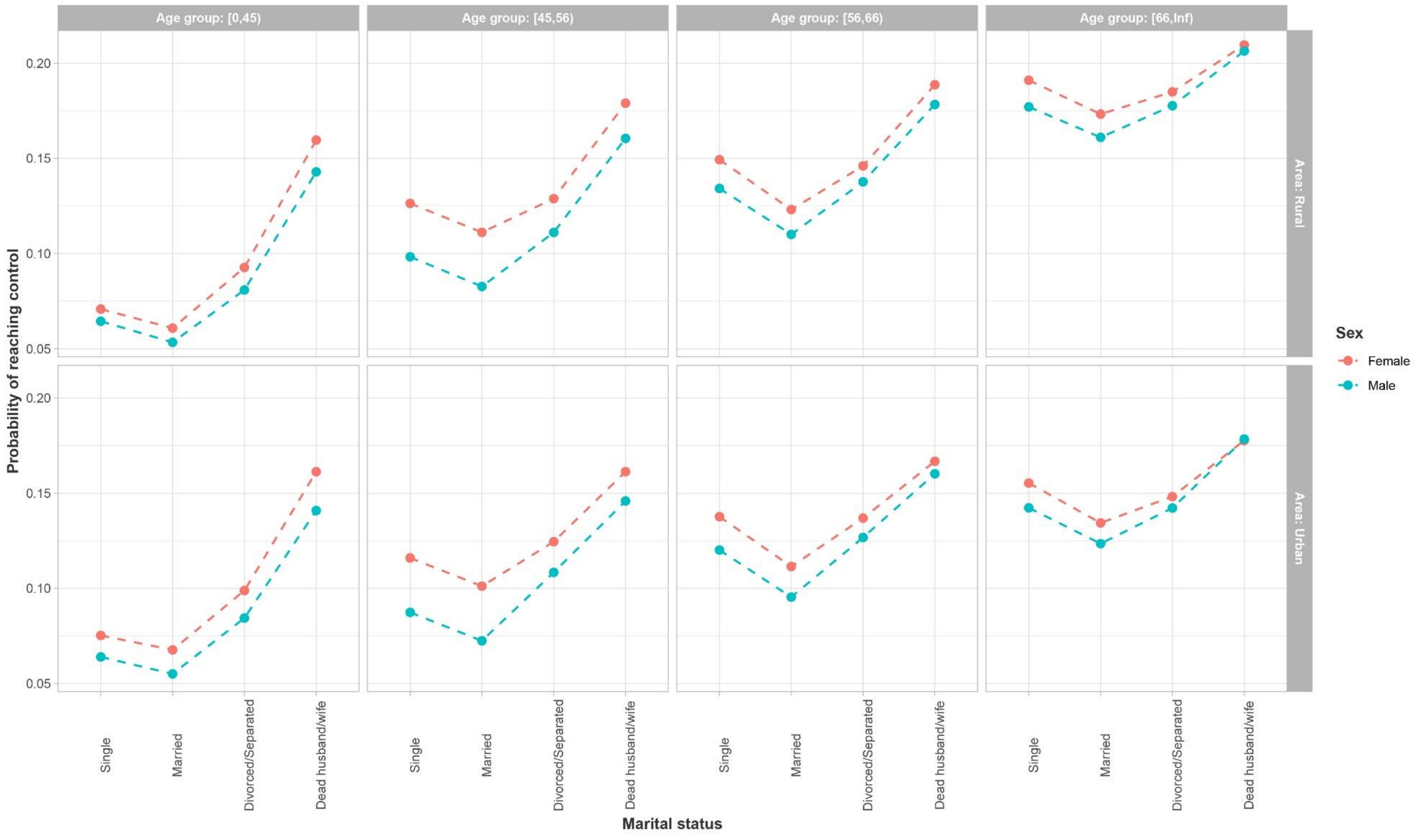

Supplemental figure S21 – Interactions between age, sex, body mass index, and diabetes mellitus for screening

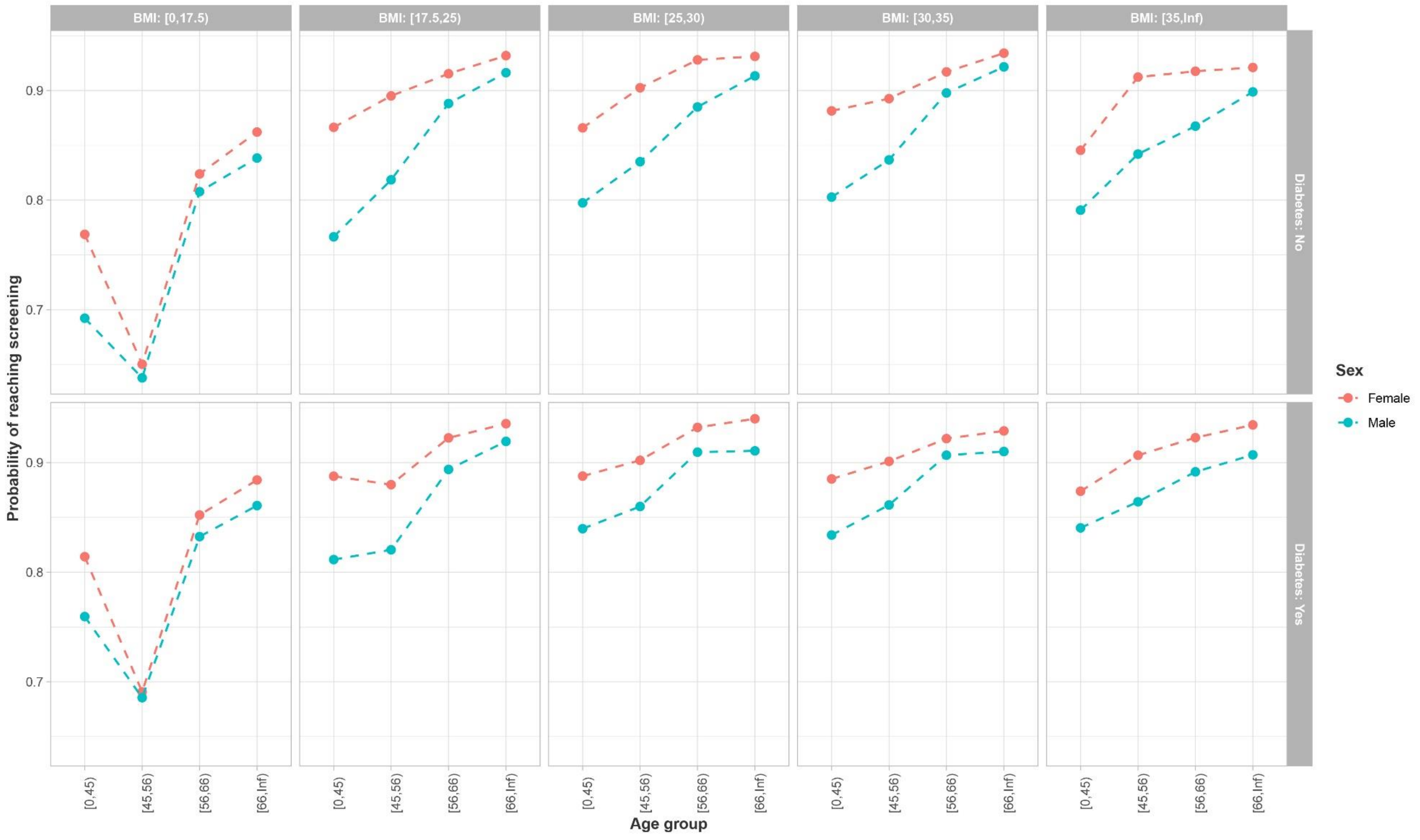

Supplemental figure S22 – Interactions between age, sex, body mass index, and diabetes mellitus for diagnosis

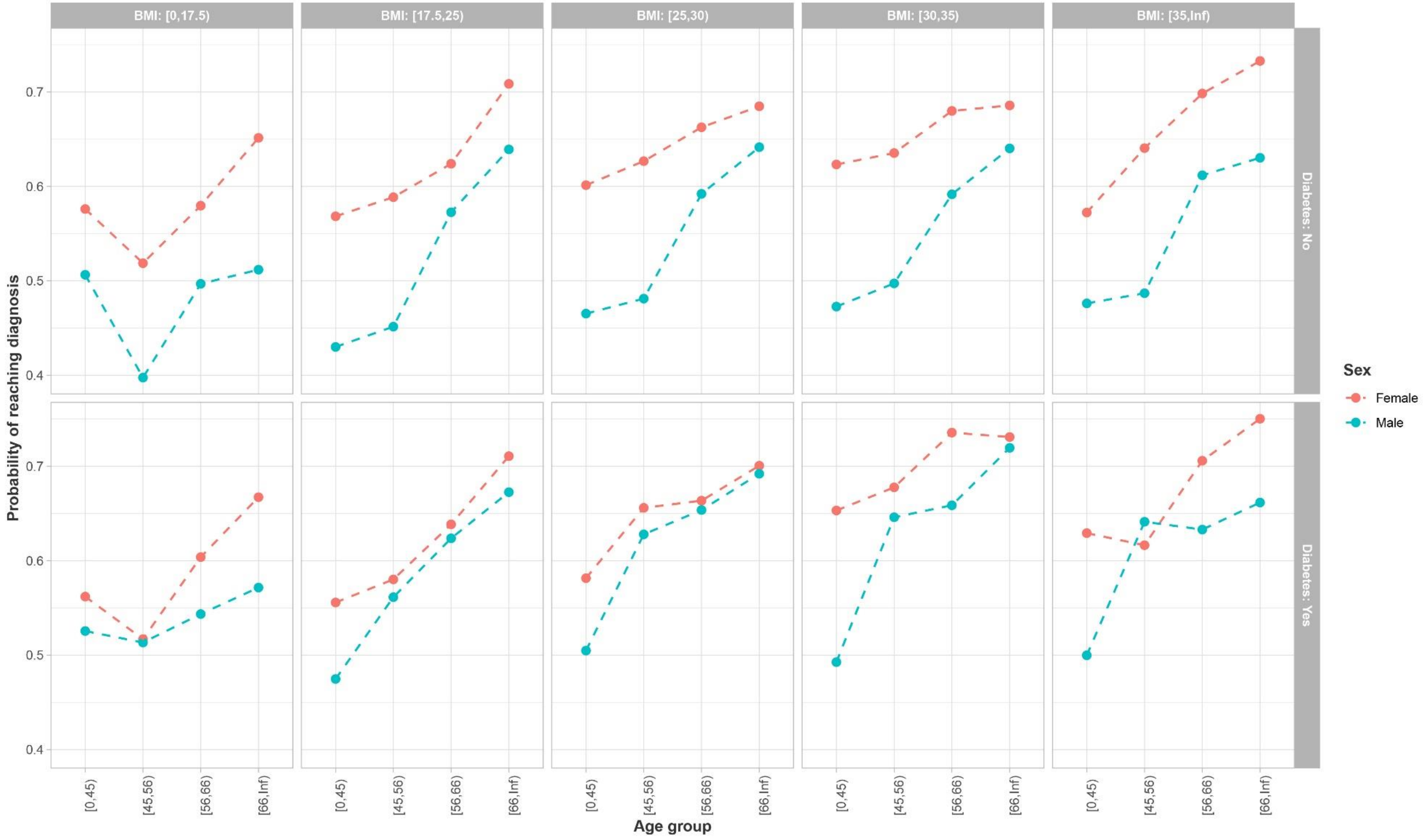

Supplemental figure S23 – Interactions between age, sex, body mass index, and diabetes mellitus for treatment

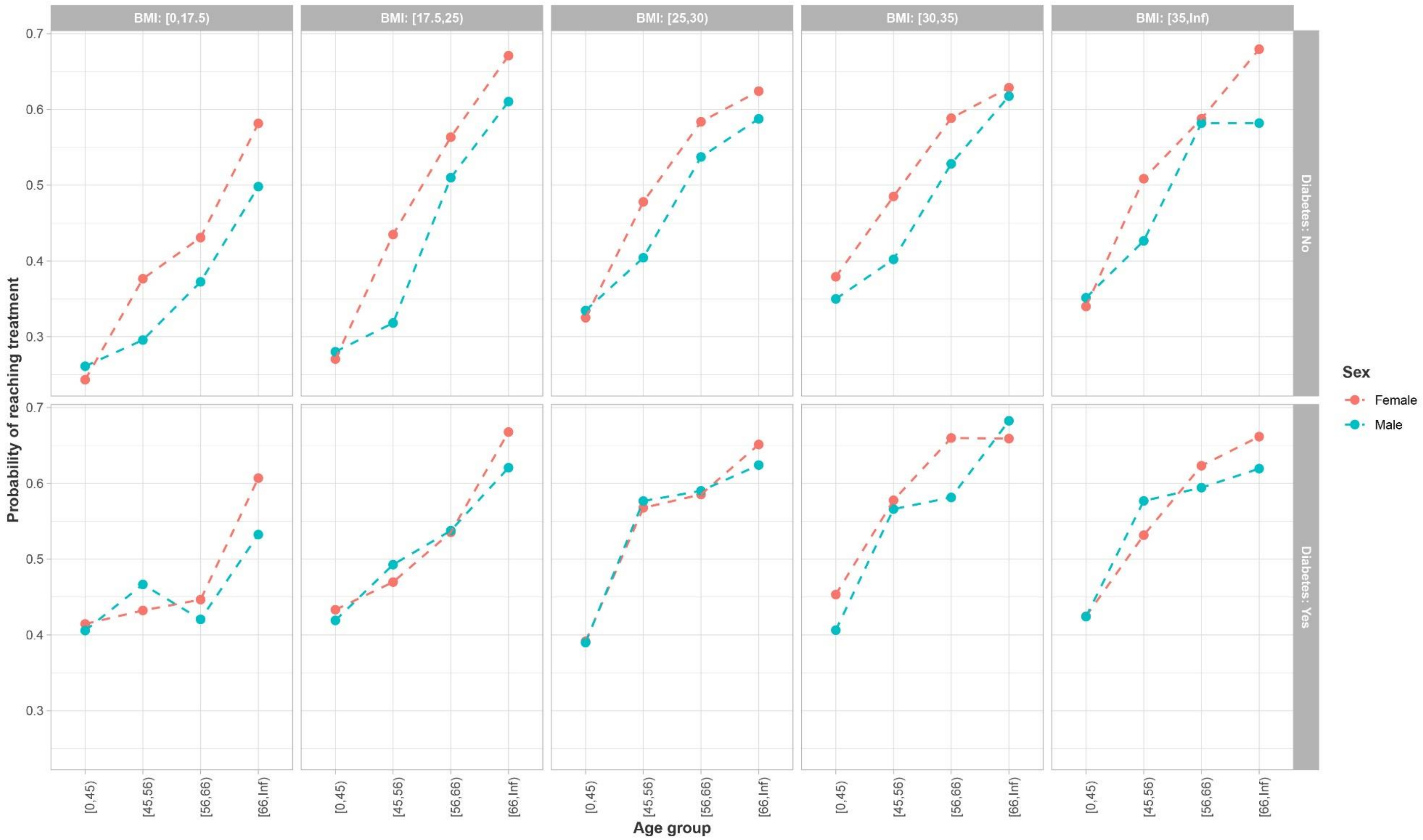

Supplemental figure S24 – Interactions between age, sex, body mass index, and diabetes mellitus for control

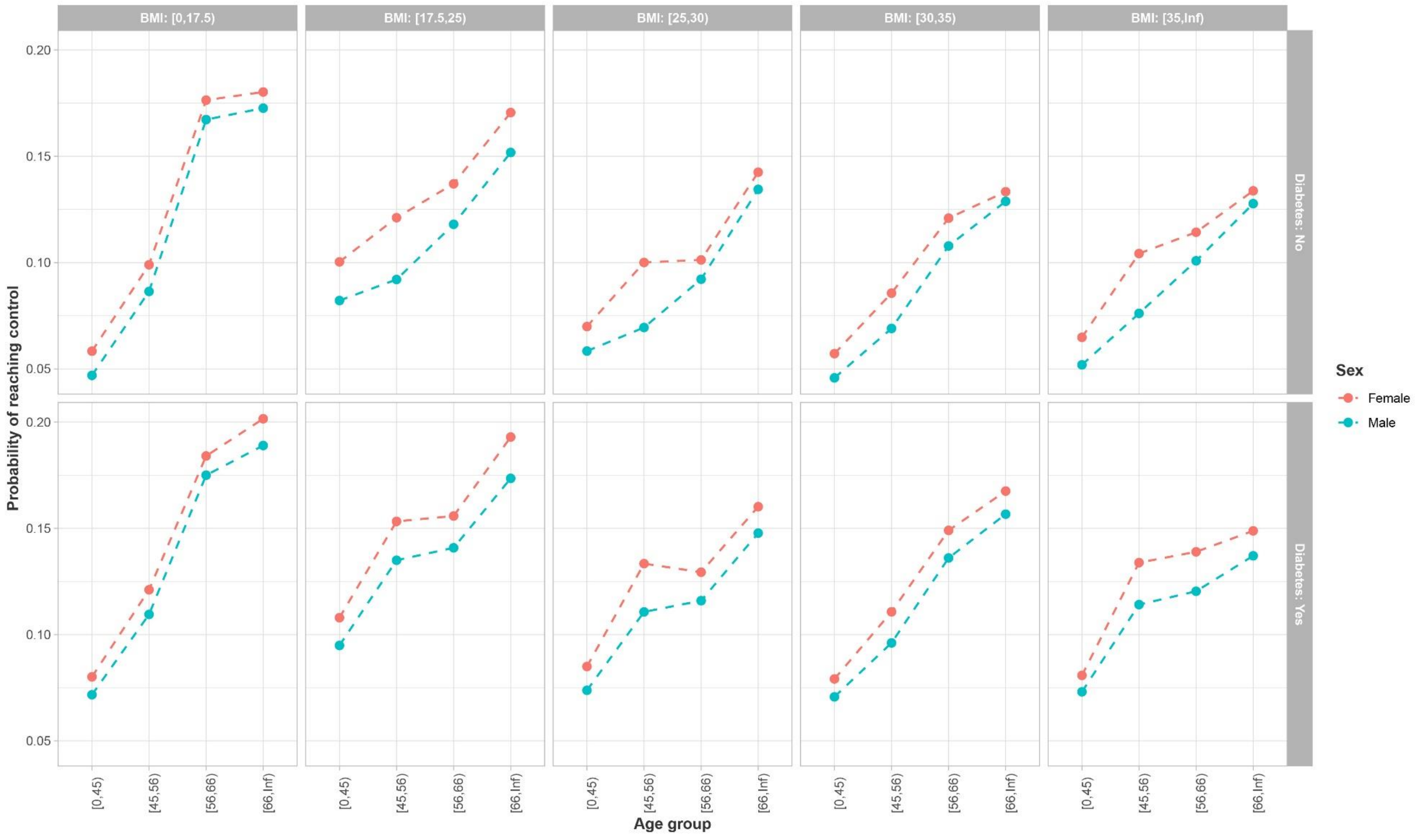

Supplemental figure S25 – Interactions between age, sex, body mass index, and smoking for screening

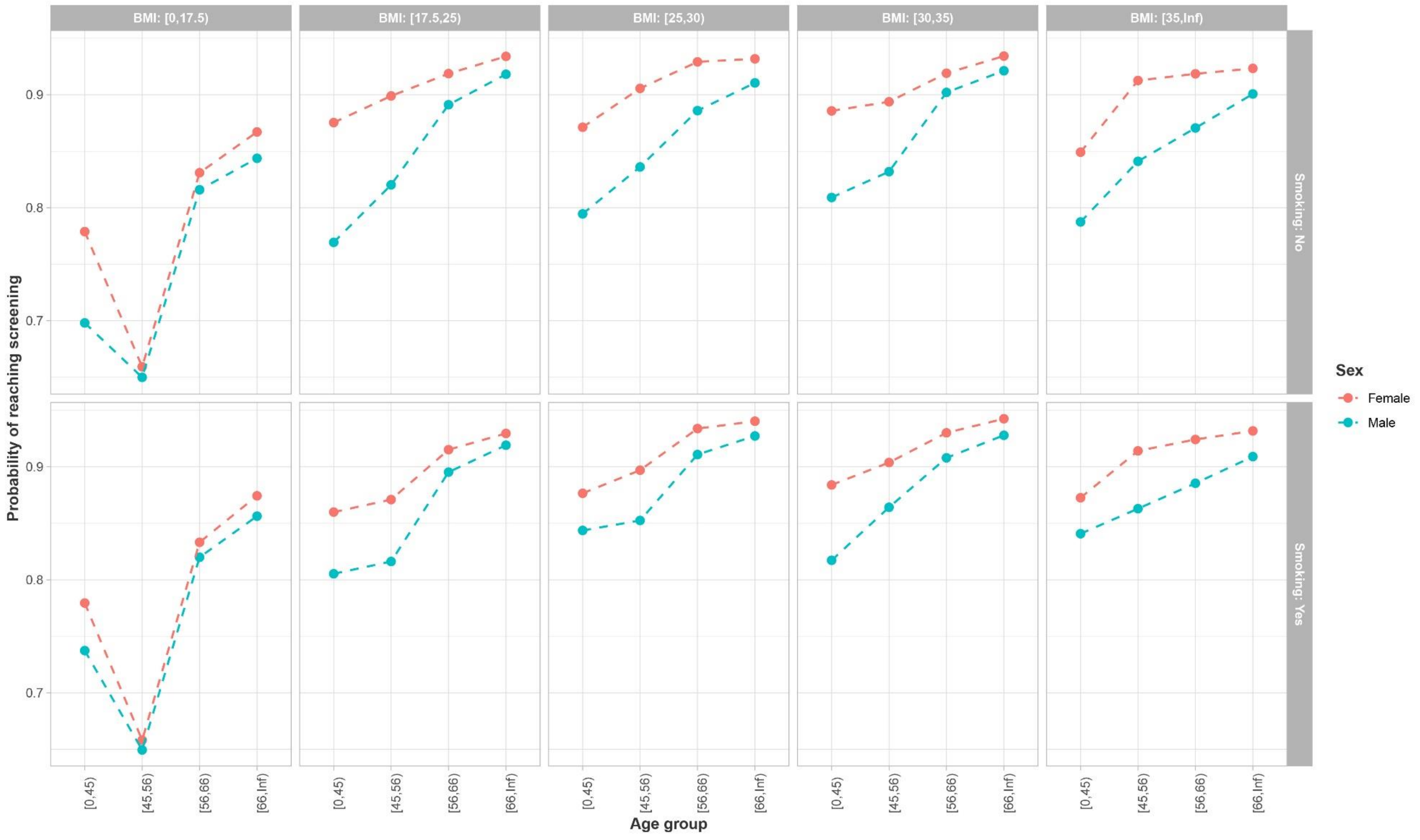

Supplemental figure S26 – Interactions between age, sex, body mass index, and smoking for diagnosis

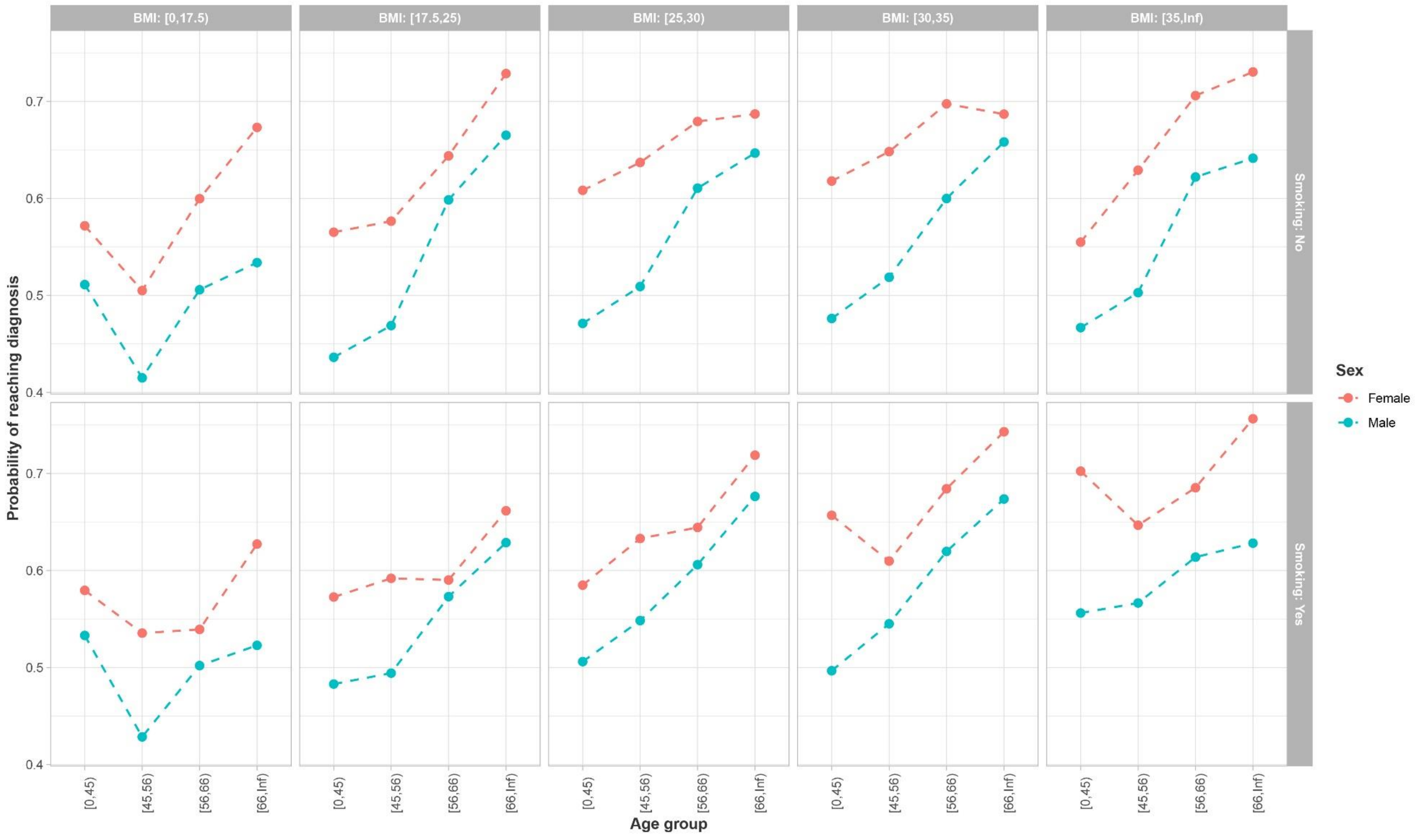

Supplemental figure S27 – Interactions between age, sex, body mass index, and smoking for treatment

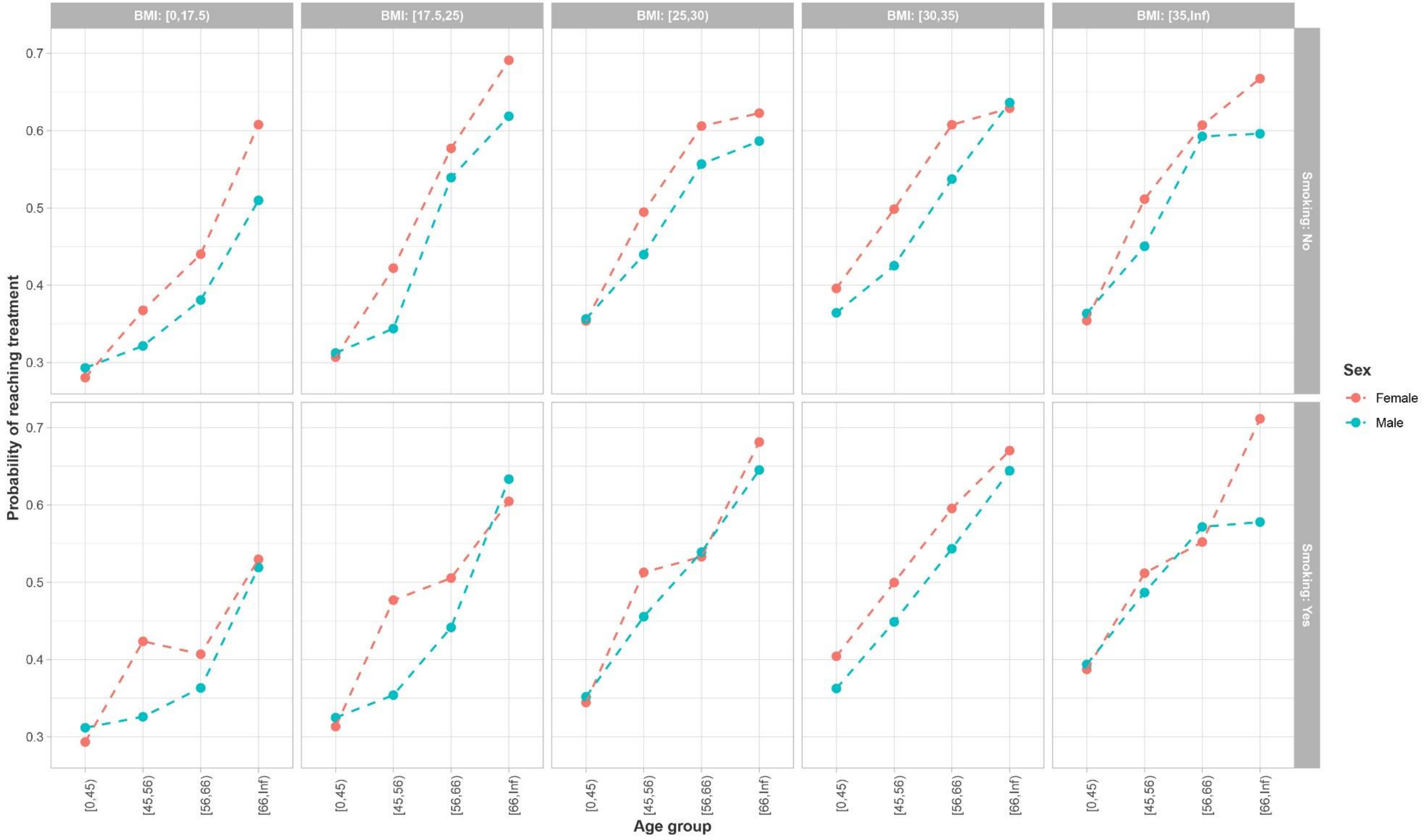

Supplemental figure S28 – Interactions between age, sex, body mass index, and smoking for control

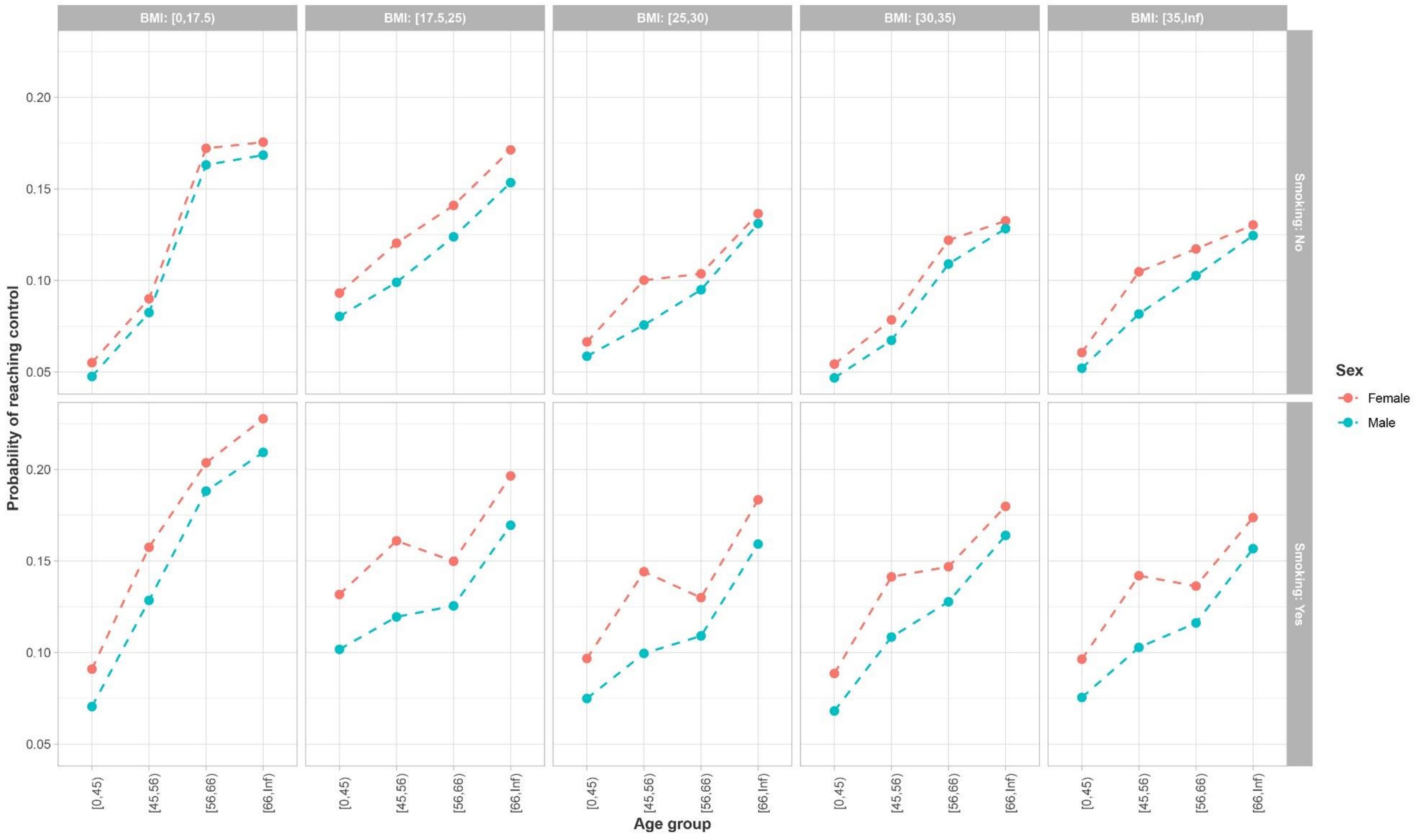

Supplement: S1 File — (PDF) [file pone.0273560.s001.pdf]
