# Supplementary material for: A machine learning approach to evaluate the state of hypertension care coverage: From 2016 STEPs survey in Iran
Source: PLoS One. 2022 Sep 21;17(9):e0273560. doi: 10.1371/journal.pone.0273560 (PMC9491523; doi:10.1371/journal.pone.0273560)
Supplement: Attachment — Submitted filename: Response to Reviewers.docx [file pone.0273560.s002.docx]

Dear Professor Radfar,

Academic editor of the PLOS ONE,

We are pleased to resubmit our revised manuscript entitled “*A machine learning approach to evaluate the state of hypertension care coverage: From 2016 STEPs survey in Iran*” (Manuscript ID: PONE-D-21-32708). On behalf of our research team, I would like to thank the respected editorial office of *PLOS ONE* and the honorable reviewers for the time and effort they took in evaluating our work. We carefully addressed the insightful comments of the reviewers. The following pages include a point-by-point response to the comments with references to the changes made in the manuscript. We made sure to indicate the added value and contribution of this study, which is an in-depth analysis of the state of hypertension care in Iran—a relevant topic considering the paucity of data from LMICs in this regard. Moreover, we addressed the concerns raised about the methodology of this investigation. As instructed, we have submitted a revised version with changes marked in highlight and a second copy without any markings. We have applied PLOS ONE style requirements to our files. We declare that this study was funded by Ministry of Health and Medical Education and National Institute for Health Research (grant number: 241-93259), in the financial disclosure and funding information.

Regarding our data availability statement, it should be noted that the STEPS 2016 study is a project launched by the Iranian Ministry of Health and Medical Education of Iran (MOHME), which owns the rights to the dataset. However, interested and qualified researchers may contact the Non-Communicable Diseases Research Center (www.ncdrc.net) to access the datasets of the STEPS 2016 study. The aggregated level data are freely accessible via https://vizit.report/en/index.html. The author-generated codes can be uploaded to GitHub and be made freely available, if the journal considers this source suitable. Otherwise, we are ready to upload the code in alternative sources that the journal would suggest.

We are looking forward to receiving your kind response and decision.

Farshad Farzadfar (MD, MPH, MSc, DSc)

Non-Communicable Diseases Research Center,

Endocrinology and Metabolism Population Sciences Institute,

Tehran University of Medical Sciences, Tehran, Iran

Address: Second Floor, No.10, Jalal Al-e-Ahmad Highway, Tehran, Iran

Postal Code: 1411713137

Tel: +98-21-88631293

Email: [f–farzadfar@tums.ac.ir](mailto:f–farzadfar@tums.ac.ir)

**Reviewer #1:**

“The publication "A machine learning approach to evaluate the state of hypertension care coverage: From 2016 STEPs survey in Iran" by Tavolinejad et al. tackles an important problem of development and progression of hypertension, moreover it is driven by the low- to middle-income country data. The authors consider hypertension to be defined as blood pressure ≥ 140/90 mmHg), or reported use of anti-hypertensive medications, or a previous hypertension diagnosis. The four steps of care considered for the analysis included screening, diagnosis, treatment, or control. Using machine learning approach (random forest), the authors estimated that from 30.8% of population with hypertension, 89.7% were screened, 62.3% received diagnosis, 49.3 were treated, and 7.9% achieved control. The random forest analysis indicated that younger age, male sex, low wealth, and unmarried/divorced status were associated with a lower probability of receiving care. As this work represents a high educational value to the populations affected by hypertension as well as may serve a the learning about detection and consequences early, this potential is diminished by several major issues related to the following aspects:**”**

**Response:** Dear respected reviewer, we hereby express our gratitude for your precise evaluation of our paper. We believe your thoughtful comments have significantly improved our work, and we tried to address the major issues you kindly indicated.

“1. Although the statistical data is rich and presented clearly (Table 1), the figures in the main manuscript are not readable. On the other hand, the figures in the supplementary files are clear and of high quality. The analyses are performed with a good technical standard and are described in sufficient detail however I disagree with the usage of the word “novel” toward the random forest application in these population data.”

**Response:** Many thank for pointing out this issue. We have increased the resolution of the main manuscript figures to improve readability. However, when the editorial manager generates the manuscript PDF file, it seems to automatically reduce image quality and compress file size. To make sure that the figures are according to journal requirements, we checked image qualities with the Preflight Analysis and Conversion Engine (PACE) digital diagnostic tool, as suggested by the journal. Furthermore, we omitted the word “novel” from the manuscript, as you recommended.

“2. The authors used random forest to evaluate the features importance for each of the stages, while the post analyses of random forest include other valuable applications that I highly recommend to the authors. To do that, the authors should move beyond feature importance or probability distribution within. The study will benefit the audience if the authors consider:

“- plotting/analyzing the probability data in higher dimension to seek for hidden relationships and patterns between e.g., age, education, status, gender, etc, i.e., patterns not limited to the conclusions that are expected from linear analysis of individual data (young single male with low income having lower care). Using the four figures to support the findings seems as a very good start to more advanced analyses and more informative conclusions. In addition, potential relationships with the biological class of features (BMI, diabetes, smoking) would start showing to play a role in only a particular cohort.”

**Response:** Many thanks for this brilliant comment. As you suggested, we looked for higher dimension interactions between features in our models (for screening, diagnosis, treatment, and control). We evaluated the associations in permutations of the variables for up to five dimensions (including the outcome and four predictive features). Accordingly, we have highlighted the most notable findings, i.e., the exceptions to the trends observed in the main analysis, in our results. However, considering we had four random forest models corresponding to four steps of hypertension care cascade, and the number of variables and investigated interactions, we generated 28 plots for this purpose. It should be noted that this many figures could not be included in the manuscript, and had to be placed in the supplements. The most notable interactions were as follows:

- “The age disparity in hypertension care, with younger patients being less likely to receive appropriate care, was more pronounced in rural than urban areas, as the gap between age groups was wider in rural communities for all steps of care (Supplemental Figures S1-4)”
- “While a higher wealth index meant a higher probability of receiving care, wealth showed interactions with education and area of residence. Higher wealth did not result in enhanced screening, diagnosis, or treatment among individuals with only primary educational attainment, contrary to better educated individuals (Supplemental Figures 5-12).”
- “Among individuals with low wealth indices, those living in rural areas received better care in screening and diagnosis compared to urban communities; however, among individuals with higher levels of wealth, more appropriate care was observed in urban areas (Supplemental Figures 5-8)”
- “While females generally had better outcomes than males, an exception to this trend was observed among single individuals, as single males had a higher likelihood to be appropriately diagnosed and treated than single females (Supplemental Figures 13-20).”
- “Investigation of interactions showed that among younger age groups, underweight and normal-weight individuals had a lower probability to be screened, diagnosed or treated. In the control step, however, a higher BMI was associated with lower achievement of BP targets.”

Obviously, looking at the interaction plots for each step of care is more informative, but we tried to summarize the findings using the most notable exceptions within the confines of an original investigation manuscript.

As for the example you suggested, about young single males with low income, we should state that while single people with low income showed a lower likelihood to receive appropriate hypertension care, among this group, females had a lower level of care compared to males.

“- how do the classifiers cluster (clustering) or relate (PCA) to one another among population?”

**Response:** Many thanks for this thoughtful suggestion. We should clarify that there is no clustering/PCA analysis done between the results of the random forest classifiers since the classifiers were fitted separately for each step of care and no relation is expected or suspected to be available among them. Even with any relations, there is no proper interpretation for it since the models were fitted independently on different filters of the main data. Please kindly let us know if you believe we have misunderstood your comment, or if further changes are warranted.

“- building a predictive model from the data, perhaps for the remaining large cohort of the population not associated with hypertension.”

**Response:** We thank the honorable reviewer for this important comment. While it would be a significant contribution to build a predictive model for hypertension care, there is an important issue that prevented us from doing so: the remainder of the STEPs cohort could not be used for this purpose because it did not include hypertensive patients. Therefore, diagnosis, treatment, and control of hypertension could not be defined for this population. One would expect inherent differences between hypertensive subjects and those with normal BP, rendering this approach unproductive. An alternative would be to test the current model in future STEPs studies in Iran, but such data is not currently available and this analysis should be done in the future. We have mentioned this shortcoming in the limitations.

“3. It is unclear if step “screening” is associated with HP when introduced in the abstract.”

**Response:** Many thanks for pointing out this issue. The screening step was defined based on the history of ever having had blood pressure measured by a health-care provider. We did not consider the presence or absence of hypertension, or the measured blood pressure value for fulfillment of screening. We tried to explain this both in the abstract and the methods.

“4. Lines 90-91: is condition (I) considered when “SBP and DBP” or “SBP or DBP” are present?”

**Response:** We must clarify that the criteria, as explained in the methods and the supplementary material, considered the presence of hypertension with either SBP≥140 or DBP≥90. We are sorry if there was ambiguity in our writing. Please let us know if changes are required.

“5. Line 99: the term “screening” still doesn't explain clearly if the BP measured was ever related to HP.”

**Response:** Many thanks for pointing out this inconsistency. As explained above, the screening step was defined based on the history of ever having had blood pressure measured by a health-care provider, and the presence or absence of hypertension, or the measured blood pressure did not have a role for this step. In addition to abstract, we added this explanation to the methods.

“6. Line 117: it is unclear what authors mean by “Random forest is an ensemble model that handles categorical variables AS THEY ARE”?”

**Response:** Thank you for raising this concern. There are different algorithms that require categorical variables to be one-hot-encoded (transformed to dummy format) like shrinkage algorithms (Ridge, LASSO, Elastic-Net & etc.) in the pre-processing step. One of the advantages of algorithms like random forest is that they face the categorical variables as they are without the need to transform them to binary forms. This was a huge advantage for our data since all of our variables were categorical and that saved us some time in the pre-processing steps. We clarified this issue in the methods.

“7. Lines 118-119: authors should expand on: “...it provides appropriate and competitive predictive accuracy compared to other algorithms”. It is recommended that authors make an explicit comparison of their analyses with other machine learning approaches before stating that random forest is the best choice for it.”

**Response:** Many thanks for this thoughtful recommendation. Indeed, there are different algorithms with different variations that can be used for similar purposes, but the important thing is to choose the best among available options for the settings of the analysis based on prior knowledge and experience, especially when model comparison is not the main target of the study. Our goal was to extract knowledge (effective variables and their direction of effect). There may always be a model to have slightly better predictive accuracy but when the aim is to obtain population-based knowledge, it is wiser to choose one of the algorithms that can be good for our settings and also the one that studies showed that it usually brings competitive results. For our study and based on our data, random forest is the algorithm that requires fewer pre-processing steps and it also provides out-of-bag validation which makes the validation process less time consuming. This way, we were able to use the time and computational power to expand our hyper-parameters space for hyper-parameter tuning and obtain an even better form of a random forest. It was mentioned that random forests have competitive predictive accuracy based on previous studies. Random forests became popular for their very good out-of-box performance that can be obtained by tuning its several hyper-parameters. It was also illustrated that among popular machine learning algorithms, random forests have the least variability in their predictive accuracy while tuning. We explained this rationale in the methods.

Reference(s):

Boehmke, B., & Greenwell, B. (2019). Hands-On Machine Learning with R (1st ed.). Chapman and Hall/CRC. https://doi.org/10.1201/9780367816377

Philipp Probst, Anne-Laure Boulesteix, and Bernd Bischl. 2019. Tunability: importance of hyperparameters of machine learning algorithms. J. Mach. Learn. Res. 20, 1 (January 2019), 1934–1965.

“8. Line 119: definition of “hyper-parameters” should be put early for the readers.”

**Response:** Thank you for this thoughtful comment. Hyper-parameters are parameters involved in different machine learning algorithms to control their complexity. More complex models have less bias but may be much variable in the results because of overfitting problem while less complex models can be too shallow and have more biased results. That’s why hyper-parameters must be tuned through an appropriate validation process to create a balance in algorithms bias-variance trade-off and deliver generalizable results. We edited the methods to put this definition early for the readers.

“9. Line 131: it is unclear what authors mean by knowledge in: “...tools were used to extract knowledge”.”

**Response:** Many thanks for pointing out this inconsistency. In the machine learning field and specifically interpretable machine learning area, knowledge is referred to the information that can be obtained by interpreting results of the model. In our specific analysis, it mainly referrs to the variable importance (obtained by permutation) and the direction of effect and interactions (obtained by investigating the partial dependence values). We omitted the phrase to avoid confusion.

“10. Line 161 and further: the class of “white collar” should be specified as either clerks or workers for consistency.”

**Response:** We thank you for pointing out this problem. We changed white-collar workers to white-collar clerks throughout the manuscript. We used “workers” only for blue-collar workers.

“11. Lines 184-185: The sentence “Very low (MPP=0.86) and low (MPP=0.87) wealth indices were associated with a decreased probability of being screened.” is unclear.”

**Response:** Many thanks for bringing this to our attention. We edited this sentence accordingly.

“12. It is suggested that, given the longitudinal data/steps present, the authors should analyze the longitudinal data on additional level; one idea could be to look at the trends in features' importance while moving along the 4 steps. More informative analysis could include population models for the subpopulations pre-classified with the machine learning tools.”

**Response:** Many thanks for this thoughtful and brilliant comment. We evaluated the probability of comparison between the four steps of care; however, because results for each step is derived from a separate model, our statistical team advised against making comparisons for feature importance across the care cascade. Although we could compare probabilities, e.g., comparing the probability of screening and treatment for single individuals, it would not be a reliable comparison. As you correctly suggested, this type of analysis would be more informative; however, it would require a longitudinal data for which a longitudinal care cascade model can be defined. As we have mentioned in the limitations, the cross-sectional design of the STEPs survey is a limitation of our study.

“In summary, the study should be enhanced by computational analyses and graphical representation of the results, including, yet not limited to those suggested in #2 and #12. If these additional analyses are added, the manuscript could be submitted to the follow-up review”

**Response:** Dear respected reviewer, we appreciate your time and effort, and your thoughtful and kind evaluation of our manuscript. We believe your brilliant comments helped improve our paper; however, there were instances where we could not perform the suggested analyses or perhaps, where we might have misunderstood your recommendations. While we hope the changes are satisfactory, please let us know if further revisions are required.

**Reviewer #2:**

“The manuscript presents a Random Forest based machine learning approach to evaluate the state of hypertension care coverage through a population-based data from Iran. Unfortunately, the paper falls short in bringing any fresh insights to the readers. Most of the causes and recommendations made by the authors are already known to the community, and it is unclear what new insight machine learning is offering, if any. Bulk of the paper is dedicated to reporting what the tool running the Random Forest model outputs, without adequate deeper dive. There is little detail on handling the data itself, as what was done to pre-process, normalize and standardize the data prior to feeding it to the machine learning model. Given the understandable lack of availability of the dataset in public domain, it is useful to create credibility around the analysis through rigorous statistics and figures. The paper lacks to explain as what the predictions mean in context of the data as well as the field itself. On what could be a very valuable dataset to analyse, I am afraid that there is a need for extended analysis and deeper interpretation of results, both in terms of machine learning model as well as for the hypertension care coverage”

**Response:** Dear respected reviewer, we thank you for your thoughtful evaluation of our paper. Thanks to your comments and those of other reviewers, we have extended our results and our interpretation of findings. A higher-dimension analysis of interactions was added to the results, to better evaluate the association of features with hypertension care. We have added the most notable interactions, i.e., those which presented exceptions to trends observed in the main analysis, to our results and discussion, and revised our conclusion, to improve our presentation of our data and its relevance. However, considering we had four random forest models corresponding to four steps of hypertension care cascade, and the number of variables and investigated interactions, we generated 28 plots for this purpose. It should be noted that this many figures could not be included in the manuscript, and had to be placed in the supplements.

As mentioned in the manuscript, nationally representative data about the current state of hypertension care in LMICs, especially in Iran, is sparse, and an in-depth analysis of this issue is relevant. For this purpose, we decided to use ensemble learning, which is a superior approach to conventional regression models and can extract more information from the data. As such, we tried to explain the analytic method that was used as much as possible. Regarding the methodologies used for STEPs 2016 data, explanation of sampling, data handling, processing, normalization, and standardization is previously published and appropriately referenced in the methods. It should be noted that pertinent aspects of STEPs methodology are dully explained in the methods. While the STEPS 2016 data are not openly available due to restrictions by the Iranian Ministry of Health and Medical Education of Iran (MOHME), interested and qualified researchers may contact the Non-Communicable Diseases Research Center (www.ncdrc.net) to access the datasets of the STEPS 2016 study. The aggregated level data are freely accessible through https://vizit.report/en/index.html.

Our most notable findings, as summarized in the paper, are: 1- Younger individuals, especially those living in rural areas or patients without other cardio-metabolic risk factors such as obesity, have a lower chance of receiving appropriate care for hypertension, not only for screening, but even when hypertension is diagnosed. 2- Males generally received less appropriate care for hypertension than females. 3- Having low socio-economic status, being single or divorced, or having blue-collar occupations or being self-employed was associated with lower levels of hypertension care. These findings highlight the gaps in current care coverage, and should be considered in future health-care policies.

We hope these revisions are satisfactory. We ask you to kindly let us know if further revisions seem necessary.

**Reviewer #3:**

“The authors present a very well-written and important paper that helps assess the reasons for the discontinuation in the care pathway through the diagnosis, treatment and control of hypertension in Iran. For that they use the random forest algorithm and conclude that younger adults, men, unmarried or divorced people and those with lower socio-economical status are more at risk to not receive treatment and achieve control of their blood pressure.

“Overall the paper was clear and presented the findings in an understandable fashion. I have two main suggestions to be discussed with the authors in two different domains.”

**Response:** Dear respected reviewer, we hereby thank you for your thoughtful review of our manuscript.

“(A) Guideline Selection and Phenotyping Algorithm

“1. Firstly, I would like to understand the reason for the selection of the older AHA guideline (2017) since in 2020 there was the release of a new version. The new guideline document can be found in the following weblink: https://www.ahajournals.org/doi/10.1161/HYPERTENSIONAHA.120.15026. In it, there are some different recommendations for the essential and optimal control of blood pressure.”

**Response:** Many thanks for this thoughtful comment. The main reason for using older recommendation for BP levels was that the data was from STEPs 2016. As the newer recommendations were not applied in practice in 2016, we could not reliably use new BP thresholds. Moreover, we tried to take a conservative level to increase the reliability of findings across steps of hypertension care. lastly, our methodology in selecting BP cut-offs and defining steps of care is similar and comparable to other studies in this field (e.g., DOI: 10.1016/S0140-6736(19)30955-9)

“2. Also, according to the author's phenotyping algorithm, hypertensive patients had SBP >= 140 and DBP >= 90 and controlled hypertensive SBP < 130 and DBP < 80. This leaves a gap in the participants who had SBP in between 130-139 and 80-89 (the new guideline covers that gap). I suggest that a closer look is taken into that.”

**Response:** We thank you for this important and brilliant comment. As explained above, the main reason for not choosing the criteria in newer guidelines was that the data was gathered in 2016. Regarding the gap you mentioned, it should be noted that the definition of hypertension was not solely based on SBP≥140 or DBP≥90 mmHg. Two other criterions were	prior diagnosis of hypertension, and use of blood pressure lowering medications. Importantly, patients who achieved the criteria for control of blood pressure should have been diagnosed or treated for hypertension, otherwise, this low blood pressure could not have been attributed to appropriate health-care.

“3. Again, regarding the guideline, usually blood pressure is measured in more than one encounter or with out-of-office techniques to discard white coat and masked hypertension. I do understand the cross-section nature of the STEPs study and the lack of longitudinal data. However, I believe this should be mentioned in the limitations as well.”

**Response:** Many thanks for pointing out this issue. As you correctly suggested, this is a limitation in our study. Thanks to your comment, we have added this to the limitations.

“(B) Algorithm Selection and Hyper-Parameter Tuning

“1. The use of the random forest algorithm is quite interesting and it is indeed powerful. However, there is another class of ensemble algorithms that usually outperforms random forest called gradient boosting machines (examples are XGBoost and LightGBM) which generally use decision trees. In that aspect, I would like to hear from the authors the reason for opting for random forest instead of one of those algorithms.”

**Response:** Many thanks for this brilliant comment. Boosting algorithms are really powerful and they usually outperform other algorithms in their class but they are less intuitive and more computationally demanding. Random forests are less demanding and easier to implement while bringing competitive results with less variability regarding the predictive accuracy. Random forests come with out-of-bag validation, which saves a lot of time in the hyper-parameter tuning process and this way we can use the time and computational power to expand the hyper-parameter space and obtain a better form of the random forest. Since the aim of our study was to reveal effective factors and interaction plus their direction of effect, a more straightforward but competitive algorithm based on our settings was random forest. We tried to emphasize this rationale in the methods.

Reference(s):

Boehmke, B., & Greenwell, B. (2019). Hands-On Machine Learning with R (1st ed.). Chapman and Hall/CRC. https://doi.org/10.1201/9780367816377

Philipp Probst, Anne-Laure Boulesteix, and Bernd Bischl. 2019. Tunability: importance of hyperparameters of machine learning algorithms. J. Mach. Learn. Res. 20, 1 (January 2019), 1934–1965.

“2. Lastly, it was not clear to me how the hyper-parameter tuning was executed. This is a very important step and it is good practice to have a separated validation set for that. It would be beneficial to understand the methodology used by the authors and have it thoroughly explained in the paper or in the supplement for better judgment of the results and also increased reproducibility”

**Response:** Many thanks for raising this issue. We tried to include a detailed description about the hyper-parameters tuning process in the second paragraph of *methods/statistical modeling*. It should be noted that there was no separate test set since there were no competing algorithms to compare their best and tuned forms on that test set. The whole data was used as the validation set in the random forest’s hyper-parameter tuning through out-of-bag validation process to make sure the final model is generalizable. Please let us know if further explanation of details seems necessary.
