# Supplementary material for: A machine learning approach to evaluate the state of hypertension care coverage: From 2016 STEPs survey in Iran
Source: PLoS One. 2022 Sep 21;17(9):e0273560. doi: 10.1371/journal.pone.0273560 (PMC9491523; doi:10.1371/journal.pone.0273560)
Supplement: Attachment — Submitted filename: Response to Reviewers.docx [file pone.0273560.s003.docx]

Dear Professor Radfar,

Academic editor of the PLOS ONE,

We are pleased to resubmit our revised manuscript entitled “*A machine learning approach to evaluate the state of hypertension care coverage: From 2016 STEPs survey in Iran*” (Manuscript ID: PONE-D-21-32708R1). On behalf of my colleagues, I would like to thank the esteemed editors and staff of *PLOS ONE* for consideration of our work. As instructed, we have submitted a revised version with tracked changes and a second copy without any markings. The following pages include a point-by-point response to the comments.

In response to the insightful comment of Reviewer #3, we have explained that the selected blood pressure thresholds were used to best reflect the state of care in Iran at the time the STEPs survey was performed. Moreover, these cut-offs are widely used in other low- and middle-income countries (LMICs), and are comparable to other available data from LMICs. We note the trade-off in selecting thresholds, which would lead to underestimation or overestimation of hypertension prevalence, diagnosis, treatment, and control. We have tried to explain our rationale more clearly, and we note in the manuscript that these thresholds may not be in total agreement with the most recent guidelines. We would appreciate any specific recommendation about changing blood pressure thresholds that could improve our paper.

Please kindly note a change we have made to the author affiliations in this revision. Furthermore, we have added two references (numbers 15 and 35) to the manuscript. As you instructed, we have checked and ensured that our references list is complete and correct. The journal requirements also stated “*If you have cited papers that have been retracted, please include the rationale for doing so in the manuscript text, or remove these references and replace them with relevant current references*”. We are not sure if this was an automated message or was in reference to a specific issue with our manuscript. We did not find a retracted article in our reference list, but please kindly let us know if there is a reference we should recheck and address.

Please do not hesitate to contact me if we need to provide any additional information about this submission. We are looking forward to receiving your kind response and decision.

Farshad Farzadfar (MD, MPH, MSc, DSc)

Non-Communicable Diseases Research Center,

Endocrinology and Metabolism Population Sciences Institute,

Tehran University of Medical Sciences, Tehran, Iran

Address: Second Floor, No.10, Jalal Al-e-Ahmad Highway, Tehran, Iran

Postal Code: 1411713137

Tel: +98-21-88631293

Email: [f–farzadfar@tums.ac.ir](mailto:f–farzadfar@tums.ac.ir)

**Reviewer #1:**

“Thanks to the Authors for addressing all the comments and explaining the unclear statements. The article is now recommended for publishing.”

**Response:** Dear respected reviewer, we thank you for your time and your constructive comments, which have improved our paper.

**Reviewer #3:**

“I have one last point that needs addressing. Overall the paper was clear and presented the findings in an understandable fashion. Most of the recommendations from the previous review were followed.
“1) Guideline Selection and Phenotyping Algorithm
“Items A and C were answered satisfactorily. However, point B is still missing:
“B. Also, according to the author's phenotyping algorithm, hypertensive patients had SBP >= 140 and DBP >= 90 and controlled hypertensive SBP < 130 and DBP < 80. This leaves a gap in the participants who had SBP between 130-139 and 80-89 (the new guideline covers that gap). I suggest that a closer look is taken into that (check lines 91-95).”

**Response:** Dear respected reviewer, we thank you for your precise and constructive comment. We apologize for we did not appropriately address one of your comments in the previous letter.

Your point is completely accurate. There is a gap in blood pressure values for definition of hypertension and control, which can be eliminated by reducing the BP cut-off for defining hypertension/high-normal BP to ≥130/80, or even by increasing the target BP for control to <140/90.

The main reason we have chosen the 140/90 threshold for hypertension is we wanted to evaluate health-care system coverage at the time of the study. We had reservations about using a lower threshold, since we were concerned it would not make an accurate simulation of the state of care and would not provide a reliable estimation of the factors associated with care, as most health-care providers may not have labeled hypertension and considered pharmacological treatment for the group with office measurements at 130-139/80-89 (high-normal BP). Our rationale was that this would underestimate the rate of treatment and control. Furthermore, the conventional cut-off of 140/90 is widely used in other low- and middle-income countries—as demonstrated by databases used in the following study: https://doi.org/10.1161/CIRCULATIONAHA.120.051620.

Importantly, it should be noted that having a BP measurement ≥140/90 was not our only criteria for hypertension definition. We also used hypertension diagnosis by health-care provider, or drug-history of BP-lowering medications. Therefore, a patient with BP<140/90 who had one of the above criteria could still be considered in the hypertensive population. Such patients would then be classified as achieving control if they had fulfilled both diagnosis and treatment steps, and had BP <130/80.

As for the control cut-off, we used 130/80 as the target because it is an optimum value for most patients for reducing adverse outcomes, and it is recommended by recent guidelines.

Ultimately, we understand that choosing any cut-off for definition of hypertension and for control would result in specific limitations. Especially since we do not have longitudinal data with multiple measurements (a point that you have thoughtfully mentioned). Thanks to your comment, we have tried to explain our rationale for choosing these numbers more clearly. In brief, these cut-offs were used because they most accurately reflect the state of care in Iran, and in most LMICs. Moreover, as several population-based studies evaluating hypertensive patients in LMICs have used similar cut-offs, this approach would increase the comparability of our results with those of others. We have also added a phrase to limitations to make this clear to readers.

We hope these explanations are satisfactory. Please kindly let us know if you think there is another specific approach that would improve this study.

“2) Algorithm Selection and Hyper-Parameter Tuning

“All the items were answered satisfactorily. In the future, the implementation of algorithms such as XGBoost and LightGBM is currently as easy as RF (there are open-source packages and libraries in Python), and they could potentially be tried out.”

**Response:** Many thanks for your insightful and expert comment. This would be a great direction for future studies.
